# Medicines for Malaria Venture COVID Box: a source for repurposing drugs with antifungal activity against human pathogenic fungi

**DOI:** 10.1590/0074-02760210207

**Published:** 2021-11-08

**Authors:** Rodrigo Almeida-Paes, Iara Bastos de Andrade, Mariana Lucy Mesquita Ramos, Marcus Vinícius de Araújo Rodrigues, Vinícius Alves do Nascimento, Andréa Reis Bernardes-Engemann, Susana Frases

**Affiliations:** 1 Fundação Oswaldo Cruz-Fiocruz, Instituto Nacional de Infectologia Evandro Chagas, Laboratório de Micologia, Rio de Janeiro, RJ, Brasil; 2 Universidade Federal do Rio de Janeiro, Instituto de Biofísica Carlos Chagas Filho, Laboratório de Biofísica de Fungos, Rio de Janeiro, RJ, Brasil

**Keywords:** antifungal activity, COVID Box, drug repurposing, pathogenic fungi

## Abstract

**BACKGROUND:**

Treatment of mycoses is often ineffective, usually prolonged, and has some side effects. These facts highlight the importance of discovering new molecules to treat fungal infections.

**OBJECTIVES:**

To search the Medicines for Malaria Venture COVID Box for drugs with antifungal activity.

**METHODS:**

Fourteen human pathogenic fungi were tested against the 160 drugs of this collection at 1.0 µM concentration. We evaluated the ability of the drugs to impair fungal growth, their fungicidal nature, and morphological changes caused to cells.

**FINDINGS:**

Thirty-four molecules (21.25%) presented antifungal activity. Seven are antifungal drugs and one is the agricultural fungicide cycloheximide. The other drugs with antifungal activity included antibiotics (n = 3), antimalarials (n = 4), antivirals (n = 2), antiparasitcs (n = 3), antitumor agents (n = 5), nervous system agents (n = 3), immunosuppressants (n = 3), antivomiting (n = 1), antiasthmatic (n = 1), and a genetic disorder agent (n = 1). Several of these drugs inhibited *Histoplasma capsulatum* and *Paracoccidioides brasiliensis* growth (15 and 20, respectively), while *Fusarium solani* was not affected by the drugs tested. Most drugs were fungistatic, but niclosamide presented fungicidal activity against the three dimorphic fungi tested. Cyclosporine affected morphology of *Cryptococcus neoformans*.

**MAIN CONCLUSIONS:**

These drugs represent new alternatives to the development of more accessible and effective therapies to treat human fungal infections.

Fungal diseases may vary from superficial, skin mycoses to invasive life-threatening infections.^1^ The incidence of fungal infections is increasing worldwide,^2^ which is a result of the emergence of new fungal pathogens driven by climatic changes^3^ or environmental disturbances caused by human activities,^4^ as well as the increasing number of at-risk patients for these infections.^5^ Most patients with fungal infections live at poverty areas, which makes diagnosis difficult and therefore impairs their real importance.^6^

The armamentarium of approved drugs available to treat fungal infections include, among other few drugs, the polyenes, azoles, flucytosine, and the echinocandins.^7^ The polyenes, which include amphotericin B, are available from more than 70 years and remain the main alternative to treat invasive and systemic infections. It binds to the ergosterol leading to the formation of pores in the fungal membrane.^8^ The azoles, which include fluconazole, itraconazole, and posaconazole, inhibit the 14α-sterol-demethylase, altering the functionality of fungal membrane.^9^ Flucytosine is an antimetabolite agent that disrupts protein synthesis and must be used in combination with other antifungal drugs, due to the risk of acquired resistance.^10^ The echinocandins were the last approved antifungal drug class that acts on the fungal cell-wall, inhibiting the biosynthesis of β-(1,3)-D-glucan.^11^

Despite the increasing burden of worldwide fungal infections, the therapeutic arsenal to combat them has not expanded at the same rate.^12^ In addition, the emergence of several species of drug resistant fungi complicates the management of mycoses with the currently available antifungal drugs.^13^ In last years, few substances were approved as new antifungals and less than a dozen of antifungal agents are in clinical phases of development.^14^ The process to develop new pharmaceutic substances is expensive and it can take up to 10 years for a drug to reach the pharmacies. Moreover, most of developed drugs never get approval for human use due to failures on any of the several phases of clinical studies.^15^

All these difficulties on the development of new drugs became evident with the emergence of the severe acute respiratory syndrome coronavirus 2 (SARS-COV-2), the agent of the ongoing coronavirus disease 2019 (COVID-19) pandemic.^16^ To overcome them, several repurposing studies for this disease were proposed.^17^ Drug repurposing is a method of drug development where substances already studied for other diseases, and often approved by some regulatory agencies, are redirected to treat a new disease or other medical conditions.^18^ In this context, the Medicines for Malaria Venture (MMV) initiative developed a collection of candidate molecules for COVID-19 repurposing (https://www.mmv.org/mmv-open/covid-box). MMV recommends a screening of the compounds at 1 µM and the confirmation of the activity of the compounds. For antifungal activity, minimal inhibitory and fungicidal concentrations, selectivity of the drug, and potential synergism with known antifungal drugs are usually tested.^19^

Hospitalised patients infected with COVID-19 are at-risk for invasive fungal infections.^20^ Moreover, there are reports of patients co-infected with SARS-COV-2 and some pulmonary endemic fungi^21^ ^,^ ^22^ or fungal agents of nosocomial pneumonia.^23^ ^,^ ^24^ Therefore, it would be advantageous the repurposing of drugs with potential SARS-COV-2 activity that are also effective against fungi that can complicate the evolution of COVID-19. The goal of this study was to search the MMV COVID Box for substances with antifungal activity against human pathogens and their effects on fungal growth and morphology.

## MATERIALS AND METHODS

*The Medicines for Malaria Venture COVID Box* - The product development partnership Medicines for Malaria Venture (MMV, Geneva, Switzerland) kindly supplied the drug collection COVID Box. It comprises 160 different substances, either marketed drugs or compounds under research/clinical development, from 19 distinct drug classes, with recognised or predicted activity against SARS-CoV-2. The Supplementary data I depicts a full list of molecules present in this drug collection. MMV provides the COVID Box as two 96-well plates containing 10 μL/well of 10 mM compound solutions in dimethylsulfoxide (DMSO), except for the compound piperaquine, which is provided at 2 mM. Dilutions of the drugs were performed to a final drug concentration of 1 mM using dimethylsulphoxide (Sigma-Aldrich, Co., St. Louis, MO, USA), as recommended by MMV. Columns 1 and 12 of each plate were used for negative (culture medium only) and positive (culture medium and inoculum) fungal growth controls, respectively. All plates were stored at −80ºC until their use in the experiments described below.

*Fungal strains and growth conditions* - Fourteen different fungal species were tested against the substances present in the COVID Box. They comprise yeasts, moulds, as well as dimorphic fungi from the three major fungal phyla that cause human infections. Table I presents the list of strains used in this study and the respective diseases that they cause. The filamentous fungi were maintained during the experiments through monthly subcultures on potato dextrose agar (Becton, Dickinson and Company, Sparks, NV, USA) at 25ºC. Yeasts were subcultured on Sabouraud dextrose agar (Becton, Dickinson and Company), 25ºC, at a weekly basis. The yeast form of the dimorphic fungi *Histoplasma capsulatum*, *Paracoccidioides brasiliensis*, and *Sporothrix brasiliensis* was obtained by weekly subcultures on the following media at 35ºC: ML-Gema agar [Mueller Hinton Broth (Difco), 2.1g; Agar, 1.0g; Dextrose, 2.0g; L-cysteine hydrochloride, 0.2g; Hen egg yolk, 15 mL], Fava-Neto [0.3% (w/v) proteose peptone (Isofar, Duque de Caxias, RJ, Brazil); 1% (w/v) peptone (Titan Biotech Ltda., Rajasthan, India); 0.5% (w/v) beef extract (Liofilchem, Roseto degli Abruzzi, TE, Italy); 0.5% (w/v) sodium chloride (Sigma-Aldrich, Co.); 0.5% (w/v) yeast extract (Becton, Dickinson and Company, Sparks, MD, USA), 4% (w/v) glucose (Neon Comercial Ltda., Suzano, SP, Brazil), 1.8% (w/v) agar (Becton, Dickinson and Company)], or Brain Heart Infusion (Becton, Dickinson and Company), respectively.

**TABLE I:** Fungal strains used in this study

Fungal species	Strain number^*^	Phyllum	Morphology	Disease
*Aspergillus fumigatus*	ATCC 204305	Ascomycota	Filamentous	Aspergilosis
*Candida haemulonii*	CFP 967	Ascomycota	Yeast	Candidiasis
*Candida krusei*	ATCC 6258	Ascomycota	Yeast	Candidiasis
*Candida parapsilosis*	ATCC 22019	Ascomycota	Yeast	Candidiasis
*Cryptococcus neoformans*	ATCC 208821 (H99)	Basidiomycota	Yeast	Cryptococcosis
*Fonsecaea pedrosoi*	CFP 791	Ascomycota	Filamentous	Chromoblastomycosis
*Fusarium solani*	ATCC 36031	Ascomycota	Filamentous	Hyalohyphomycosis
*Histoplasma capsulatum*	ATCC 26032 (G217B)	Ascomycota	Dimorphic	Histoplasmosis
*Neoscytalidium dimidiatum*	CFP 803	Ascomycota	Filamentous	Cutaneous mycosis
*Neoscytalidium hyalinum*	CFP 804	Ascomycota	Filamentous	Cutaneous mycosis
*Paracoccidioides brasiliensis*	Pb18	Ascomycota	Dimorphic	Paracoccidioidomycosis
*Rhizopus oryzae*	CFP 781	Zygomycota	Filamentous	Mucormycosis
*Rhodotorula mucilaginosa*	CFP 898	Basidiomycota	Yeast	*Rhodotorula* fungemia
*Sporothrix brasiliensis*	CBS 120339	Ascomycota	Dimorphic	Sporotrichosis

*: ATCC - American type culture collection; CBS: centraalbureau voor Schimmelcultures; CFP: coleção de fungos patogênicos.

*COVID Box screening for antifungal drugs* - All compounds present in the MMV COVID Box were further diluted as recommended by the manufacturer for the screening against the above-mentioned fungi. We used a methodology, based on the Brazilian Committee on Antimicrobial Susceptibility Testing (BrCAST) guidelines for antifungal susceptibility, standardised by our group to test another MMV drug collection.^19^ In brief, compounds were tested at a final concentration of 2 μM in 100 μL of RPMI 1640 medium, with phenol red, with L-glutamine, and without sodium bicarbonate (Sigma-Aldrich, Co), buffered with morpholine propanesulfonic acid (Vetec Química Fina Ltda, Rio de Janeiro, RJ, Brazil) at pH 7.0, and supplemented for a final 2% glucose (Neon Comercial Ltda). Diluted drugs were distributed in 96-well plates (Kasvi Ltda, São José dos Pinhais, PR, Brazil). For preparation of the fungal inoculum, the strains were grown as described above and then suspended in RPMI 1640 and vortexed, with the suspension adjusted to 1 × 10^6^ cells/mL. This suspension was further diluted 1:10 in RPMI 1640 and then 100 μL of the fungal inoculum was added to each well containing the compounds, generating a final working inoculum density of 5 × 10^4^ cells/mL, drug concentrations of 1 µM, and a final DMSO concentration of 0.5% in each well, including controls. Plates were incubated at 35ºC for 12-96 h, depending on the fungal species tested. Plates were visually inspected daily and, when sufficient fungal growth was observed in the control wells, all wells without fungal growth were recorded. The screening was performed in triplicate, and only drugs that consistently inhibited fungal growth on all experiments were considered as hits.

*Microscopic evaluation* - Immediately after the identification of hit drugs as described above, 100 µL of each hit well was transferred to a microcentrifuge tube. Centrifugation was conducted in an Eppendorf 5415C centrifuge at 5,000 g for 10 minutes. Pellets were suspended in 10 µL lactophenol cotton blue (Sigma-Aldrich) and observed under a light microscope at 1,000 magnification. The same procedure was repeated in control wells, without addition of drugs. Fungal morphology in hit wells was compared with the morphology of the fungus grown in control wells.

*Fungicidal activity* - To check whether the hit drugs only inhibited fungal growth or killed the fungi, immediately after the identification of hit drugs as described above, 5 µL of each hit well were subcultured on Sabouraud dextrose agar and incubated at 25ºC until 21 days, when we checked for fungal growth in the plates. If the plates presented fungal growth, the hit drug was classified as fungistatic. If no fungal growth was detected, hit drugs were classified as fungicidal. This experiment was performed in triplicate, and only drugs that consistently killed the fungi in all tests were considered fungicidal.

*Time kill assay* - Selected drugs with fungicidal activity were used in this assay. Fungal inocula were prepared at 1 × 10^4^ cells/mL in RPMI 1640, pH 7.0, as described above. Drugs were added at a 1 µM concentration and cultures were incubated at 35ºC. Control conditions consisted in the same fungal inoculum in RPMI 1640 supplemented with 0.5% DMSO, the same concentration present in test cultures. Colony forming unit (CFU) counts were performed immediately after inoculum and drug mixture, as well as after 6, 24, 48, 72, and 96 h of incubation, through 10-fold serial dilutions in phosphate buffered saline of the fungal cultures supplemented or not with the drugs tested. Time-kill curves were generated using the GraphPad Prism 8.4.2 software and comparisons between control and test curves were performed using the Student t-test.

*Literature investigation* - Original, peer-reviewed articles, published in English, Portuguese, or Spanish up to December 31st, 2020 were searched in the PubMed database (https://pubmed.ncbi.nlm.nih.gov/) to check whether the hit molecules detected in this study presented described antifungal activity against other fungal species not included here. The search strategy consisted in the combination of the trivial name of the hit drugs with the expressions “antifungal activity” or “fungus”. Known antifungal drugs used to treat patients with mycoses were not included in this investigation. The resulting articles were reviewed and papers without clear methodologies of drug repurposing approaches against fungi were excluded.

## RESULTS

**Fig. 1:**
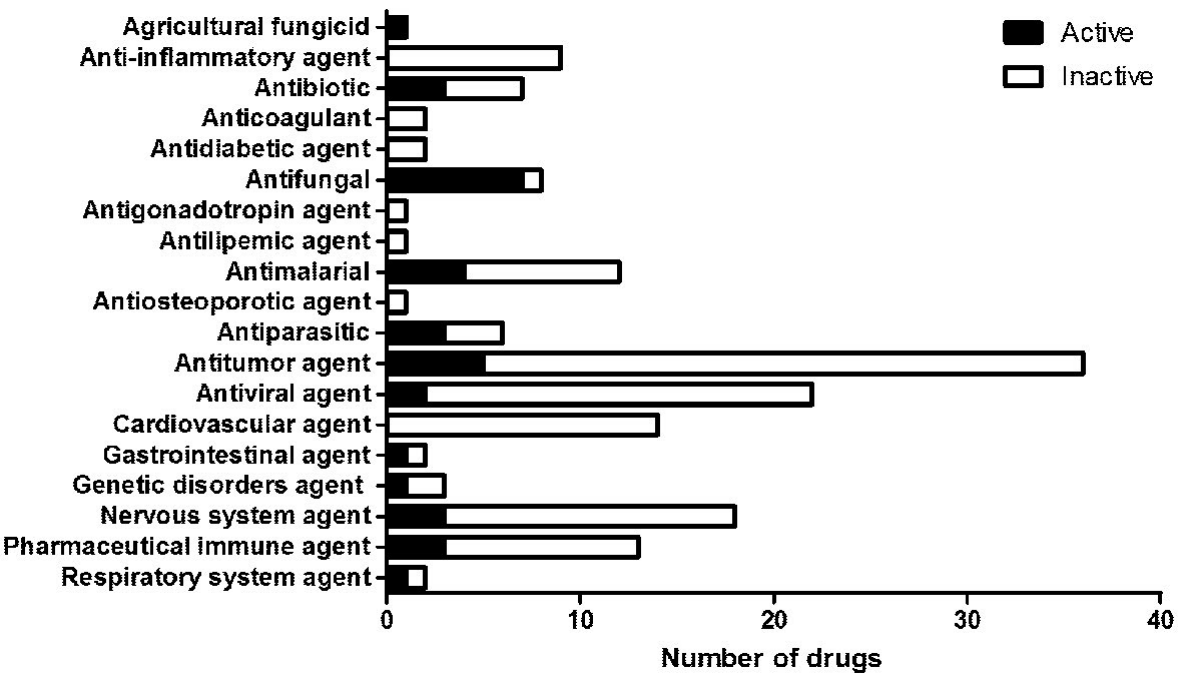
antifungal activity of drugs present in the Medicines for Malaria Venture COVID Box (MMV COVID Box), according to their original indication. Nineteen drug classes are present in the drug collection. Known antifungal drugs served as controls of antifungal activity. Bars represent the number of active and inactive drugs against any of the studied fungi.

*The MMV COVID Box has drugs presenting antifungal activity* - Among the 160 substances present in the MMV COVID Box, 34 were able to inhibit the growth of at least one of the studied fungi. Fig. 1 shows the distribution of drugs with antifungal activity according to their original indication. Seven of these active substances are known antifungal drugs (ketoconazole, itraconazole, ravuconazole, terconazole, posaconazole, fluconazole, and anidulafungin) and one is the agricultural fungicide cycloheximide. The other drugs presenting antifungal activity included three antibiotics; four antimalarials; two antivirals, one of them with anti-HIV activity; three antiparasitc agents, five antitumor agents, two of them used to treat leukemia; three nervous system agents with antipsychotic, antispasmodic, or anti-schizophrenia properties; three pharmaceutical immune agents with immunosuppressant properties; one gastrointestinal agent with antivomiting properties; one respiratory system agent with antiasthmatic properties; and one genetic disorder agent used to treat Hutchinson-Gilford progeria syndrome.

**Fig. 2:**
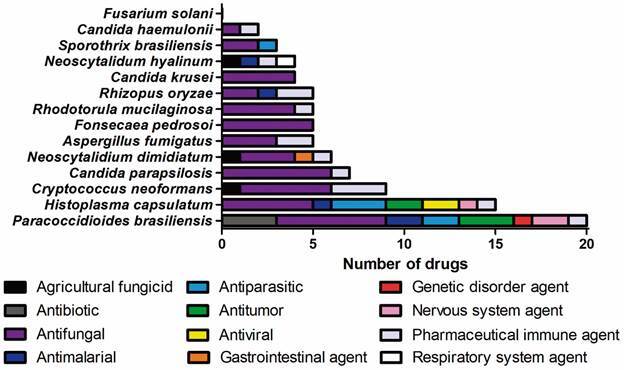
susceptibility of 14 human pathogenic fungi against the 34 drugs with antifungal activity during the screening of the Medicines for Malaria Venture COVID Box (MMV COVID Box), according to their drug class. Activity of known antifungal drugs were as expected for each fungal species.

*Different pathogenic fungi have distinct responses against the MMV COVID Box drugs* - Fig. 2 shows the susceptibility of the 14 studied fungi against the 34 drugs that presented antifungal activity during the screening of the MMV COVID Box, according to their drug class. Briefly, the dimorphic endemic fungi *H. capsulatum* and *P. brasiliensis* were the most susceptible fungi. They were inhibited by 15 and 20 different drugs, respectively. The yeast *Candida krusei* and the filamentous dematiaceous fungus *Fonsecaea pedrosoi* were inhibited only by antifungal drugs. The filamentous fungi *Fusarium solani* was not inhibited by any of the studied substances at 1 µM, even the antifungal drugs presented in the studied collection.

**Fig. 3:**
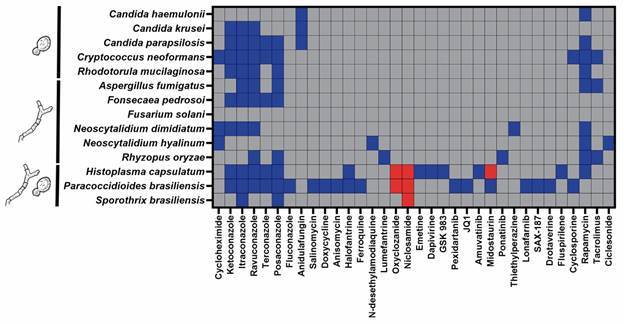
drugs with antifungal activity detected in the current study. The fungistatic (blue squares), fungicidal (red squares) or inactive nature (gray squares) of each drug against 14 human pathogenic fungi is presented. Known antifungal drugs served as controls of antifungal activity.

*Most of MMV COVID Box effective drugs are fungistatic* - Fig. 3 shows the hit drugs detected in the current study, together with their fungistatic, fungicidal or inactive nature against the 14 human pathogenic fungi included in this study. Niclosamide was fungicidal against the three dimorphic fungi tested. The three antibiotics with antifungal activity (salinomycin, anisomycin, and doxycycline) were effective only against *P. brasiliensis*, while the two antivirals detected as hits were only active against *H. capsulatum*, both also presenting fungistatic activity against this species. The antimalarial drug lumefantrine was fungistatic only against the mucormycete *Rhyzopus oryzae*, while another antimalaric drug, N-Desethylamodiaquine, presented fungistatic activity against *Neoscytalidium hyalinum*, which was also the only fungus inhibited by the antiasthmatic drug ciclesonide. Different drugs, thiethylperazine and rapamycin, inhibited its closest relative *Neoscytalidium dimidiatum*. In fact, immunosuppressive drugs, especially rapamycin, were fungistatic against most studied fungi.

**Fig. 4:**
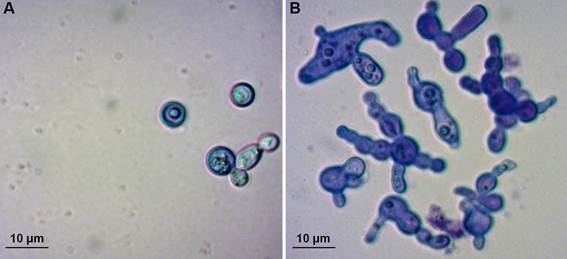
changes in *Cryptococcus* neoformans surface morphology caused by cyclosporine A. Drug-free control (A) and 1 µM cyclosporine (B) cultures were observed with lactophenol cotton blue in a light microscope. Bars: 10 µm.

*Cyclosporine affects Cryptococcus neoformans morphology* - In general, the hit drugs did not affect fungal morphology, since morphologies in hit wells were similar to that observed in control wells for all drug/fungus combinations, except for cyclosporine and *C. neoformans*. In the presence of this immunosuppressant drug, *C. neoformans* loses its regular spherical morphology and presents with an irregular surface (Fig. 4).

**Fig. 5:**
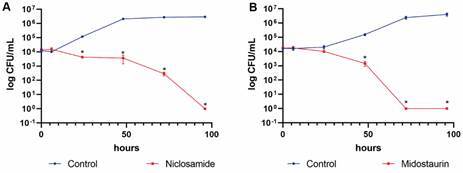
time-kill curves of selected drugs with off-label antifungal activity. Growth curves of Sporothrix brasiliensis with 1 µM niclosamide (A) and Histoplasma capsulatum with 1 µM midostaurin (B) are presented. *p < 0.05 relative to the drug-free control fungal culture.

*Fungicidal drugs differentially affect the dimorphic fungi* - To check whether the fungicidal effects of niclosamide were similar among the three dimorphic fungi, time kill curves of *H. capsulatum*, *P. brasiliensis*, and *S. brasiliensis* in the presence of this drug were constructed. After six hours of incubation with niclosamide, *H. capsulatum* and *P. brasiliensis* were completely killed by the drug, while for *S. brasiliensis*, fungal cells were slowly killed during 96 h of incubation with this drug (Fig. 5A). An additional time-kill curve of *H. capsulatum* with midostaurin, another fungicidal hit drug against this fungus. Contrary to niclosamide, midostaurin-induced *H. capsulatum* killing was slow, taking 72 h to present complete fungal killing (Fig. 5B).

**Fig. 6:**
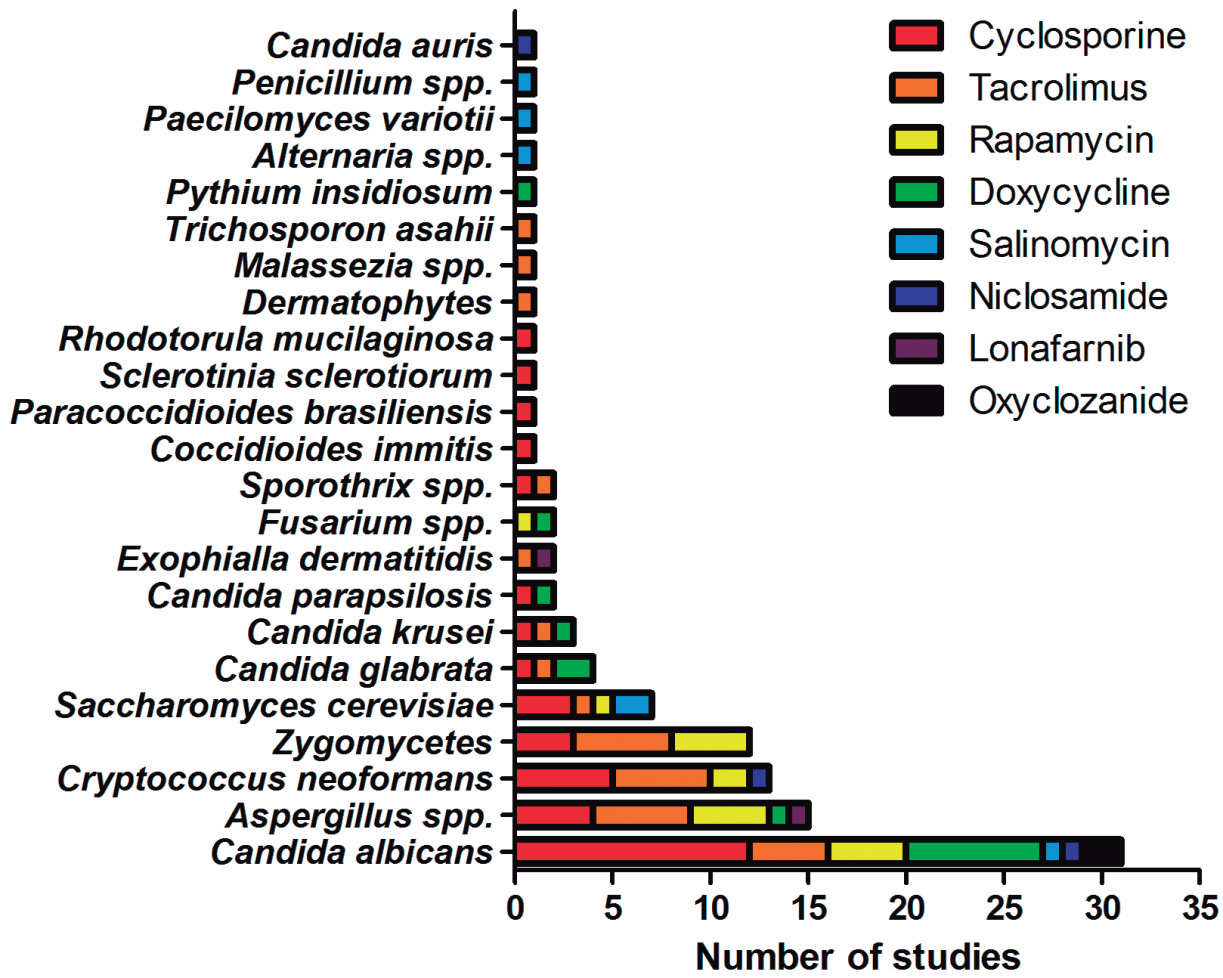
literature reports of antifungal activity among the 26 drugs with off-label antifungal activity detected in this study. Eighteen drugs did not have reported off-label antifungal activity to date. Bars represent the number of published papers in the studied databases reporting in vitro or *in vivo* studies with eight drugs and several fungi.

*Reports of antifungal activity of hit molecules* - A literature search revealed 68 studies (Supplementary data II) reporting antifungal activity for the non-antifungal drugs herein identified as hits. Among the 26 hit drugs with off-label antifungal activity detected in the present study, eight (30.8%) already were described as able to inhibit fungal growth: cyclosporine, tacrolimus, rapamycin, doxycycline, salinomycin, niclosamide, lonafarnib, and oxyclozanide. Therefore, to the best of our knowledge, 18 drugs are herein described as presenting antifungal activity for the first time: anisomycin, halofantrine, ferroquine, N-desethylamodiaquine, lumefantrine, emetine, dapivirine, GSK 983, pexidartinib, JQ1, amuvatinib, midostaurin, ponatinib, thiethylperazine, SAX 187, drotaverine, fluspirilene, and ciclesonide. Fig. 6 shows the number of studies describing the susceptibility of 23 groups/species of fungi against these drugs. Thirty-nine studies presented minimal inhibitory concentrations for these drugs against planktonic fungal cells (Table II). It is interesting to note that 46 studies (67.6%) report synergism between these candidate repurposed drugs and other molecules, mostly approved antifungal drugs used in the treatment of human patients.

**TABLE II:** Minimal inhibitory concentrations (MIC) of eight repurposed drugs against planktonic fungal cells

Drug	Fungus	MIC range	
		µg/mL	µM
Ciclosporin A	*Aspergillus* spp.	1.56 - 50	1.3 - 41.6
	*Candida* spp.	6.25 - > 512	5.2 - > 426
	*Coccidioides immitis*	1	0.83
	*Cryptococcus* spp.	0.39 - 5	0.32 - 4.16
	Mucorales	< 1 - 16	< 0.83 - 13.3
	*Saccharomyces cerevisiae*	> 16	> 13.3
	*Sporothrix* spp.	1	0.83
Doxycycline	*Candida* spp.	> 200	> 450
	*Fusarium*	> 16	> 36
	*Pythium insidiosum*	1 -16	2.25 - 36
Lonafarnib	*Aspergillus* spp.	> 16	> 25
	*Exophiala dermatitidis*	> 16	> 25
Niclosamide	*Crytococcus*	0.05	0.169
Oxyclozanide	*Candida* spp.	13 - 34	32.4 - 84.7
Rapamycin	*Aspergillus* spp.	50	54.7
	*Candida* spp.	0.02 - 0.2	0.02 - 0.21
	*Cryptococcus* spp.	0.19	0.21
	Mucorales	< 1 - 100	< 1.1 - 109
Salinomycin	*Candida* spp.	> 100	> 133
Tacrolimus	*Aspergillus* spp.	0.02 - >256	0.025 - > 318
	*Candida* spp.	10 - 512	12.4 - 637
	*Cryptococcus* spp.	<0.09 - 12.5	< 0.11 - 15.5
	Dermatophytes	> 8	> 9.95
	*Exophiala dermatitidis*	> 16	> 19.9
	*Malassezia* spp.	16 - 32	19.9 - 39.8
	Mucorales	< 1 - 8	< 1.24 - 9.95
	*Saccharomyces cerevisiae*	200 - >400	249 - > 498
	*Sporothrix* spp.	1 - 2	1.24 - 2.49
	*Trichosporon asahii*	> 64	> 79.6

## DISCUSSION

The difficulties in the treatment of fungal infections with the current approved antifungal drugs,^13^ the paucity of therapeutic options for patients with COVID-19,^25^ and several reports of SARS-COV-2 and human pathogenic fungi co-infections^23^ ^,^ ^24^ ^,^ ^26^ ^,^ ^27^ make drug repurposing against both COVID-19 and mycoses an interesting approach. The strategy used in this study, a screening of a drug collection of drugs with supposed action against SARS-CoV-2, against a large collection of pathogenic fungi, lead to the discovery of 34 molecules that can be theoretically used to treat both infections. Eighteen of them were never described as active against pathogenic fungi.

The most susceptible fungi against the drugs present in the MMV COVID Box at 1 µM were *P. brasiliensis* and *H. capsulatum*. A higher susceptibility of *P. brasiliensis* compared with other fungal species was also observed when a panel of fungi was tested against the natural product curcumin.^28^ Some drugs have been described with antifungal activity against *P. brasiliensis*, including cyclosporin A.^29^ This last drug also presented antifungal activity against this species in the present study. Currently, paracoccidioidomycosis, the infection caused by *P. brasiliensis*, can be treated with a repurposed drug, the antibiotic co-trimoxazole,^30^ and in this study, three other antibiotics presented anti-*P. brasiliensis* activity. In fact, only this fungal species was affected by antibiotics presented in the MMV COVID Box. In addition, only *H. capsulatum* was susceptible to some antiviral drugs present in the MMV COVID Box. Interestingly, the anti-*Histoplasma* activity of the antivirals saquinavir and ritonavir was previously described.^31^

A new group of at-risk patients for invasive fungal infections has emerged with the COVID-19 pandemia.^20^ In fact, co-infections with SARS-CoV-2 and human pathogenic fungi such as those of the genus *Aspergillus*,^24^ *Candida*,^26^ *Fusarium*,^23^ *Rhyzopus*,^27^ among others, are reported. Unfortunately, the drugs of the MMV COVID Box did not affect most of the fungi belonging to these genera in this study. *Fusarium* species, in particular, are intrinsically resistant to most antifungal drugs and commonly acquire resistance in the clinical setting.^32^ Therefore, the results of this study confirm the resistance profile of this genus not only against traditional antifungals, but also against a large panel of bioactive drugs. Mucormycosis agents, including *R. oryzae*, are also inherent resistant to several antifungal drugs.^33^ In this study, the antimalarial lumefantrine presented fungistatic activity against *R. oryzae*. This is an aryl-amino alcohol that probably interferes with byproduct detoxification of heme degradation. Interestingly, this mucormycete is inhibited by haemofungin, a compound that also interferes with heme metabolism.^34^

As demonstrated by our literature search, it is well known that *Candida albicans* is highly inhibited by a series of drugs presented in the MMV COVID Box. This was the reason why we choose other less studied *Candida* species to use in the experiments of this study. *Candida krusei* is intrinsically resistant to fluconazole,^35^ while *Candida haemulonii* is a poorly studied, multidrug-resistant emerging pathogen.^36^ *Candida parapsilosis*, on the other hand, is traditionally susceptible to antifungal drugs, but present increasing rates of fluconazole resistance in last decades.^37^

Except for the antifungal drugs present in the MMV COVID Box, only rapamycin was able to inhibit *C. parapsilosis* and *C. haemulonii* growth at the concentration suggested by the manufacturer for the screening. This drug acts against *Mucor circinelloides* in a mechanism dependent on the FKBP12-inhibition of the TOR pathway.^38^ The inhibition of this signaling pathway appears to be the mechanism of action of this drug and its analogs, such as cyclosporin A and tacrolimus, against *Candida* and *Aspergillus*.^39^ Rapamycin, together with tacrolimus, was also able to inhibit *Aspergillus fumigatus* in the present study. Both are immunosuppressive drugs, which are molecules with known *in vitro* antifungal activity. However, the impairment these drugs cause in the host surpasses their antifungal activity, since patients that use these immunosuppressive drugs usually develop severe forms of fungal infections.^40^ Therefore, they cannot be used systemically to treat fungal infections. Repurposing of immunosuppressive drugs to treat fungal infections must involve the use of topic formulations to be used in sites where the immune response is not likely to have an impact on fungal pathogenesis, such as a rapamycin solution in a nail polish vehicle for *Neoscytalidium* onychomycosis, or the chemical modification of these molecules, yielding compounds retaining antifungal activity but with little or no activity against the host immune system.

Most of the drugs with antifungal activity identified for the first time in this study present fungistatic activity. Azoles, the currently most used antifungal class to treat human fungal infections, acting on ergosterol biosynthesis,^9^ are also fungistatic against most pathogenic fungi.^41^ The lack of morphological changes caused by hit drugs with off-label antifungal activity in the fungi studied may indicate mechanisms of action not associated with cellular apoptosis, lysis or disruption, which is in accordance with the fungistatic nature of most hit drugs discussed before. In addition, fungi were able to grow when subcultured in drug-free media after incubation with most tested drugs, including the ones with new descriptions of antifungal activity.

Niclosamide was identified in this study as fungicidal against dimorphic fungi. The mechanisms of action of this drug against cancer, bacterial, viral, and metabolic diseases include uncoupling of oxidative phosphorylation and modulation of Wnt/β-catenin, NF-κB, STAT3, mTORC1, and Notch signaling pathways.^42^ Niclosamide is an anti-helminthic drug with a short half-life and low bioavailability, which impairs their use to treat systemic infections such as histoplasmosis and paracoccidioidomycosis. However, due to its broad spectrum of activity against viruses, bacteria, and cancer cells, nanotechnology and pro-drug approaches are in development,^42^ which may represent a new option to treat mycoses caused by endemic dimorphic fungi.

Other limitations related to the pharmacokinetics, pharmacodynamics, or contraindications of drugs with antifungal activity presented in the COVID Box include low bioavailability (especially for niclosamide, drotaverine, and ciclesonide), reproductive toxicity (for midostaurin and lonafarnib), central nervous system toxicity (thiethylperazine), or pregnancy contraindication (especially for doxycycline, pexidartinib, and lonafarnib). Chemical modification or nanotechnology approaches with these drugs are encouraged for possible future clinical trials against mycotic infections with these drugs.

It is interesting to note that some drugs present in the MMV COVID Box have published data reporting an antifungal activity that was not detected in the current study. These include cyclosporine and tacrolimus activity against *C. krusei* ^43^ and *S. brasiliensis* ^44^ as well as niclosamide activity against *C. neoformans*.^45^ These differences may be related to different susceptibility profiles of the strains used, differences in drug concentrations in the assays, or differences in the methodology for drug repurposing screening. In addition, doxycycline synergistic activity with antifungal drugs was observed with *Fusarium* spp.,^46^ *C. krusei*, and *C. parapsilosis*.^47^ The mechanism of action of this synergistic effect appears to be related to an interference with iron homeostasis.^48^ Further studies of synergism between the drugs present in the MMV COVID Box and the currently used antifungal drugs may reveal new molecules with potential antifungal activity.

The number of strains of each species included in this study is indeed a limitation of the work. Since one strain of each species was tested, we cannot discard the possibility of individual strain susceptibility bias. This is particularly true for drugs presenting antifungal activity against a single fungal species, such as ciclesonide (active only against *N. hyalinum*) or fluspirilene (active only against *H. capsulatum*). Another limitation was the use of a single concentration of drugs for screening, as recommended by the manufacturer of the COVID Box. This may have led to the discard of some molecules with potential antifungal activity, since drug concentrations are critical to their fungicidal nature as well as to affect fungal growth and morphology. Further pre-clinical studies involving testing of the antifungal activity of the herein described drugs with a large number of strains and at different concentrations are necessary.

In conclusion, this work has revealed new drugs with off-label antifungal activity to serve as alternatives to the development of more accessible and effective therapies to treat human fungal infections. Unfortunately, we were not able to identify a drug with a broad-spectrum of action against several fungi. In the future, studies to enhance drug bioavailability, to reduce immunosuppressive effect of drugs while maintaining their antifungal activity, and to combine drugs should be performed with the hit drugs identified in this study.
