# Supplementary material for: Medicines for Malaria Venture COVID Box: a source for repurposing drugs with antifungal activity against human pathogenic fungi
Source: Mem Inst Oswaldo Cruz. 2021 Nov 8;116:e210207. doi: 10.1590/0074-02760210207 (PMC8577065; doi:10.1590/0074-02760210207)
Supplement: Supplementary file 1 [file 1678-8060-mioc-116-e210207-s1.pdf]

STRUCTURE	ENTITY_ID	TRIVIAL_NAME	CAS N°	CHEM_NAME	FORMULA	MW	Development status	Indication	Litterature
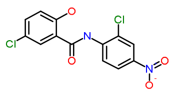	MMV003461	Niclosamide	50-65-7	5-chloro-N-(2-chloro-4-nitrophenyl)-2-hydroxybenzamide	C13 H8 Cl2 N2 O4	327,122993	Launched	Anti-infective agent - Antiparasitic	doi.org/10.1101/2020.03.20.999730
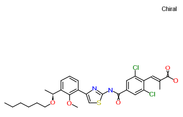	MMV1804192	Lusutrombopag	1110766-97-6	(E)-3-[2,6-dichloro-4-[[4-[3-[(1S)-1-hexoxyethyl]-2-methoxyphenyl]-1,3-thiazol-2-yl]carbamoyl]phenyl]-2-methylprop-2-enoic acid	C29 H32 Cl2 N2 O5 S	591,54578	Launched	Pharmaceutical immune agent - Thrombocytopenia	doi.org/10.1101/2020.03.20.999730
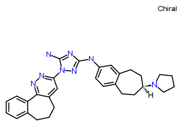	MMV1804190	Bemcentinib	1037624-75-1	1-(3,4-diazatricyclo[9.4.0.02,7]pentadeca-1(15),2,4,6,11,13-hexaen-5-yl)-3-N-[(7S)-7-pyrrolidin-1-yl-6,7,8,9-tetrahydro-5H-benzo[7]annulen-3-yl]-	C30 H34 N8	506,64456	Ph II	Antitumor agent	https://www.bergenbio.com/pipeline/bemcentinib-covid-19/
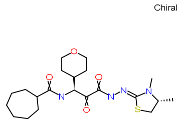	MMV1804177	ONO 5334	868273-90-9	N-[(1S)-3-[(2E)-2-[(4R)-3,4-dimethyl-1,3-thiazolidin-2-ylidene]hydrazinyl]-1-(oxan-4-yl)-2,3-dioxopropyl]cycloheptanecarboxamide	C21 H34 N4 O4 S	438,58406	Ph II, discontinued	Antiosteoporotic agent	doi.org/10.1101/2020.04.16.044016
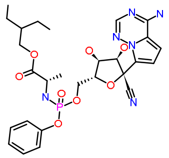	MMV1803859	Remdesivir	1809249-37-3	2-ethylbutyl (2S)-2-[[[(2R,3S,4R,5R)-5-(4-aminopyrrolo[2,1-f][1,2,4]triazin-7-yl)-5-cyano-3,4-dihydroxyoxolan-2-yl]methoxy-phenoxyphosphoryl]ami	C27 H35 N6 O8 P	602,57596	Launched	Anti-infective agent - Antiviral agent	doi.org/10.1101/2020.03.20.999730
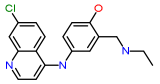	MMV659065	N-Desethylamodiaquine	79352-78-6	4-[(7-chloroquinolin-4-yl)amino]-2-(ethylaminomethyl)phenol	C18 H18 Cl N3 O	327,814994	Research	Anti-infective agent - Antimalarial	
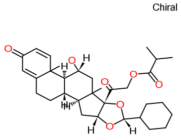	MMV1580187	Ciclesonide	126544-47-6	[2-[(1S,2S,4R,6R,8S,11R,12S)-6-cyclohexyl-11-hydroxy-9,13-dimethyl-16-oxo-5,7-dioxapentacyclo[10.8.0.02,9.04,8.013,18]icosa-14,17-dien-8-yl]-2-	C32 H44 O7	540,68756	Launched	Respiratory system agent - Antiasthmatics	doi.org/10.1101/2020.03.20.999730

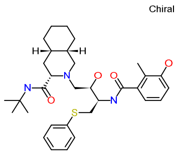	MMV637677	Nelfinavir	159989-64-7	(3S,4aS,8aS)-N-tert-butyl-2-[(2R,3R)-2-hydroxy-3-[(3-hydroxy-2-methylbenzoyl)amino]-4-phenylsulfanylbutyl]-3,4,4a,5,6,7,8,8a-octahydro-1H-	C32 H45 N3 O4 S	567,7824	Launched	Anti-infective agent - Anti-HIV agent	www.medrxiv.org/content/10.1101/2020.04.16.20068379v1.full.pdf
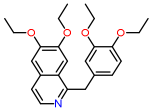	MMV003140	Ethaverine	486-47-5	1-[(3,4-diethoxyphenyl)methyl]-6,7-diethoxyisoquinoline	C24 H29 N O4	395,498991	Launched	Cardiovascular agent - Coronary vasodilator	doi.org/10.21203/rs.3.rs-23951/v1
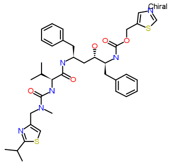	MMV637861	Ritonavir	155213-67-5	1,3-thiazol-5-ylmethyl N-[(2S,3S,5S)-3-hydroxy-5-[[(2S)-3-methyl-2-[[methyl-[(2-propan-2-yl-1,3-thiazol-4-yl)methyl]carbamoyl]amino]butanoyl]amino]-1,6-diphenylhexan-2-	C37 H48 N6 O5 S2	720,94422	launched	Anti-infective agent - Anti-HIV agent	doi.org/10.1016/j.phrs.2020.104859
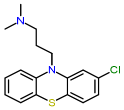	MMV001871	Chlorpromazine	50-53-3 69-09-0	3-(2-chlorophenothiazin-10-yl)-N,N-dimethylpropan-1-amine	C17 H19 Cl N2 S	318,86416	Ph II, discontinued	Antitumor agent	doi: 10.1016/j.encep.2020.04.010
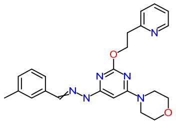	MMV1804187	Apilimod	541550-19-0	N-[(3-methylphenyl)methylideneamino]-6-morpholin-4-yl-2-(2-pyridin-2-ylethoxy)pyrimidin-4-amine	C23 H26 N6 O2	418,49154	Ph II	Antitumor agent - B-cell non-Hodgkin's lymphoma	doi.org/10.1101/2020.04.16.044016
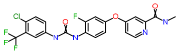	MMV1804185	Regorafenib	755037-03-7	4-[4-[[4-chloro-3-(trifluoromethyl)phenyl]carbamoylamino]-3-fluorophenoxy]-N-methylpyridine-2-carboxamide	C21 H15 Cl F4 N4 O3	482,81541	Launched	Antitumor agent	doi.org/10.21203/rs.3.rs-23951/v1
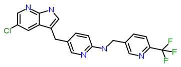	MMV1804178	Pexidartanib	1029044-16-3	5-[(5-chloro-1H-pyrrolo[2,3-b]pyridin-3-yl)methyl]-N-[[6-(trifluoromethyl)pyridin-3-yl]methyl]pyridin-2-amine	C20 H15 Cl F3 N5	417,81481	Ph II	Antitumor agent	doi.org/10.21203/rs.3.rs-23951/v1

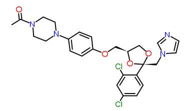	MMV637533	Ketoconazole	65277-42-1	1-[4-[4-[[rac-(2R,4S)-2-(2,4-dichlorophenyl)-2-(imidazol-1-ylmethyl)-1,3-dioxolan-4-yl]methoxy]phenyl]piperazin-1-yl]ethanone	C26 H28 Cl2 N4 O4	531,43092	Launched	Anti-infective agent - Antifungal	doi.org/10.21203/rs.3.rs-23951/v1
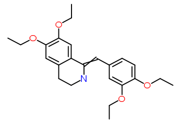	MMV852425	Drotaverine	14009-24-6 985-12-6	1-[(3,4-diethoxyphenyl)methylidene]-6,7-diethoxy-3,4-dihydro-2H-isoquinoline	C24 H31 N O4	397,50724	Launched	Nervous system agent - Antispasmodic	doi.org/10.21203/rs.3.rs-23951/v1
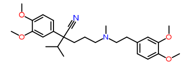	MMV002398	Verapamil	52-53-9 152-11-4	2-(3,4-dimethoxyphenyl)-5-[2-(3,4-dimethoxyphenyl)ethyl-methylamino]-2-propan-2-ylpentanenitrile	C27 H38 N2 O4	454,60162	Launched	Cardiovascular agent - Antihypertensive	doi.org/10.1016/j.apsb.2020.02.008
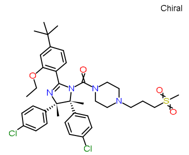	MMV1804226	R 7112	939981-39-2	[(4S,5R)-2-(4-tert-butyl-2-ethoxyphenyl)-4,5-bis(4-chlorophenyl)-4,5-dimethylimidazol-1-yl]-[4-(3-methylsulfonylpropyl)piperazin-1-yl]methanone	C38 H48 Cl2 N4 O4 S	727,78312	Ph Ib, discontinued	Antitumor agent	
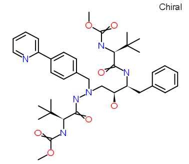	MMV659028	Atazanavir	198904-31-3	methyl N-[(2S)-1-[2-[(2S,3S)-2-hydroxy-3-[[(2S)-2-(methoxycarbonylamino)-3,3-dimethylbutanoyl]amino]-4-phenylbutyl]-2-[(4-pyridin-2-	C38 H52 N6 O7	704,85548	launched	Anti-infective agent - Anti-HIV agent	doi.org/10.1101/2020.04.04.020925.
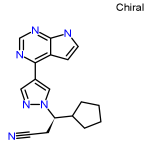	MMV1804179	Ruxolitinib	941678-49-5	(3R)-3-cyclopentyl-3-[4-(7H-pyrrolo[2,3-d]pyrimidin-4-yl)pyrazol-1-yl]propanenitrile	C17 H18 N6	306,36502	Launched	Pharmaceutical immune agent - Thrombocytopenia	doi.org/10.1101/2020.03.20.999730 doi.org/10.1016/S1473-3099(20)30132-8
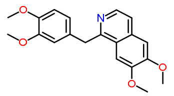	MMV002798	Papaverine	58-74-2 61-25-6	1-[(3,4-dimethoxyphenyl)methyl]-6,7-dimethoxyisoquinoline	C20 H21 N O4	339,38504	Launched	Nervous system agent - Antispasmodic	doi.org/10.21203/rs.3.rs-23951/v1

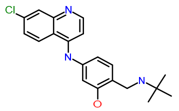	MMV000029	GSK-369796	1010411-21-8 459133-38-1	2-[(tert-butylamino)methyl]-5-[(7-chloroquinolin-4-yl)amino]phenol	C20 H22 Cl N3 O	355,868993	Ph I, stopped	Anti-infective agent - Antimalarial	doi.org/10.1101/2020.04.16.044016
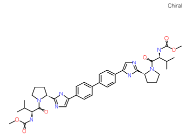	MMV1804186	Daclatasvir	1009119-64-5	methyl N-[(2S)-1-[(2S)-2-[4-[4-[4-[2-[(2S)-1-[(2S)-2-(methoxycarbonylamino)-3-methylbutanoyl]pyrrolidin-2-yl]-1H-imidazol-4-yl]phenyl]phenyl]-1H-	C40 H50 N8 O6	738,875	Launched	Anti-infective agent - Antiviral agent	en.irct.ir/trial/46463
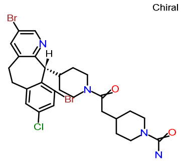	MMV658920	Lonafarnib	193275-84-2	4-[2-[4-[(2R)-6,15-dibromo-13-chloro-4-azatricyclo[9.4.0.03,8]pentadeca-1(15),3,5,7,11,13-hexaen-2-yl]piperidin-1-yl]-2-oxoethyl]piperidine-1-	C27 H31 Br2 Cl N4 O2	638,82164	Approval awaited	Genetic disorders agent - HGP syndrome	doi.org/10.21203/rs.3.rs-23951/v1
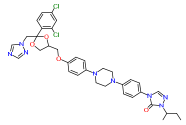	MMV637528	Itraconazole	84625-61-6	2-butan-2-yl-4-[4-[4-[4-[[2-(2,4-dichlorophenyl)-2-(1,2,4-triazol-1-ylmethyl)-1,3-dioxolan-4-yl]methoxy]phenyl]piperazin-1-yl]phenyl]-1,2,4-triazol-3-one	C35 H38 Cl2 N8 O4	705,63342	Launched	Anti-infective agent - Antifungal	clinicaltrialsregister.eu/ctr-search/trial/2020-001243-15/BE
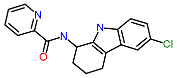	MMV690621	GSK 983	827591-02-6	N-(6-chloro-2,3,4,9-tetrahydro-1H-carbazol-1-yl)pyridine-2-carboxamide	C18 H16 Cl N3 O	325,79214	Preclinical, discontinued	Anti-infective agent - Antiviral agent	doi.org/10.1016/j.chembiol.2020.05.002
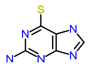	MMV662539	Tioguanine	154-42-7	2-amino-7H-purine-6-thiol	C5 H5 N5 S	167,193997	Launched	Antitumor agent - Leukemia	doi.org/10.21203/rs.3.rs-23951/v1
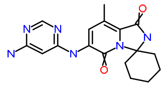	MMV1804184	Tomivosertib	1849590-01-7	6-[(6-aminopyrimidin-4-yl)amino]-8-methylspiro[2H-imidazo[1,5-a]pyridine-3,1'-cyclohexane]-1,5-dione	C17 H20 N6 O2	340,3797	Ph II	Antitumor agent	doi.org/10.1016/j.bbi.2020.05.078

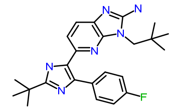	MMV690777	LY 2228820	862505-00-8	5-[2-tert-butyl-4-(4-fluorophenyl)-1H-imidazol-5-yl]-3-(2,2-dimethylpropyl)imidazo[4,5-b]pyridin-2-amine	C24 H29 F N6	420,52566	Ph II, discontinued	Antitumor agent	doi.org/10.1101/2020.05.12.091256
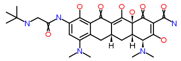	MMV1804180	Tigecycline	220620-09-7	(4S,4aS,5aR,12aR)-9-[[2-(tert-butylamino)acetyl]amino]-4,7-bis(dimethylamino)-1,10,11,12a-tetrahydroxy-3,12-dioxo-4a,5,5a,6-tetrahydro-4H-	C29 H39 N5 O8	585,64866	Launched	Anti-infective agent - Antibacterial	doi.org/10.1016/j.apsb.2020.02.008
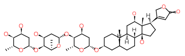	MMV002832	Digoxin	20830-75-5	3-[(3S,5R,8R,9S,10S,12R,13S,14S,17R)-3-[(2R,4S,5S,6R)-5-[(2S,4S,5S,6R)-5-[(2S,4S,5S,6R)-4,5-dihydroxy-6-methyloxan-2-yl]oxy-4-	C41 H64 O14	780,93846	Launched	Cardiovascular agent - Antiarrhythmic	doi.org/10.1101/2020.03.20.999730
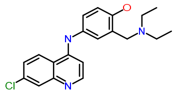	MMV000001	Amodiaquine	69-44-3 86-42-0	4-[(7-chloroquinolin-4-yl)amino]-2-(diethylaminomethyl)phenol	C20 H22 Cl N3 O	355,86118	Launched	Anti-infective agent - Antimalarial	doi.org/10.1101/2020.03.20.999730
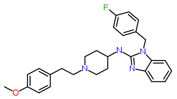	MMV003162	Astemizole	68844-77-9	1-[(4-fluorophenyl)methyl]-N-[1-[2-(4-methoxyphenyl)ethyl]piperidin-4-yl]benzimidazol-2-amine	C28 H31 F N4 O	458,57034	Withdrawn	Pharmaceutical immune agent - Antihystaminic	doi.org/10.1101/2020.04.16.044016
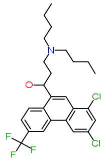	MMV000012	Halofantrine	36167-63-2 69756-53-2	3-(dibutylamino)-1-[1,3-dichloro-6-(trifluoromethyl)phenanthren-9-yl]propan-1-ol	C26 H30 Cl2 F3 N O	500,42371	Launched	Anti-infective agent - Antimalarial	doi.org/10.36877/pddbs.a0000065
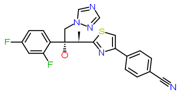	MMV1634362	Ravuconazole	182760-06-1	4-[2-[(2R,3R)-3-(2,4-difluorophenyl)-3-hydroxy-4-(1,2,4-triazol-1-yl)butan-2-yl]-1,3-thiazol-4-yl]benzonitrile	C22 H17 F2 N5 O S	437,46509	Ph II, discontinued	Anti-infective agent - Antifungal	doi.org/10.21203/rs.3.rs-23951/v1

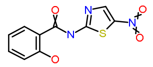	MMV1804183	Tizoxanide	173903-47-4	2-hydroxy-N-(5-nitro-1,3-thiazol-2-yl)benzamide	C10 H7 N3 O4 S	265,24528	Research	Anti-infective agent - Antiparasitic	prodrug of nitazoxanide
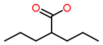	MMV003305	Valproic Acid	99-66-1	2-propylpentanoic acid	C8 H16 O2	144,213997	Launched	Nervous system agent - Anticonvulsant	www.biorxiv.org/content/10.1101/2020.03.22.002386v1.full.pdf
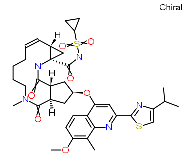	MMV1804227	Simeprevir	923604-59-5	(1R,4R,6S,7Z,15R,17R)-N-cyclopropylsulfonyl-17-[7-methoxy-8-methyl-2-(4-propan-2-yl-1,3-thiazol-2-yl)quinolin-4-yl]oxy-13-methyl-2,14-dioxo-3,13-diazatricyclo[13.3.0.04,	C38 H47 N5 O7 S2	749,93908	Launched	Anti-infective agent - Antiviral agent	doi.org/10.20944/preprints202002.0438.v1
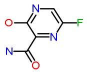	MMV688370	Favipiravir	259793-96-9	6-fluoro-3-hydroxypyrazine-2-carboxamide	C5 H4 F N3 O2	157,10256	Launched	Anti-infective agent - Antiviral agent	doi.org/10.1016/j.eng.2020.03.007
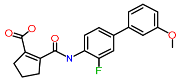	MMV1804113	Vidofludimus	717824-30-1	2-[[2-fluoro-4-(3-methoxyphenyl)phenyl] carbamoyl]cyclopentene-1-carboxylic acid	C20 H18 F N O4	355,35962	Ph II	Pharmaceutical immune agent	clinicaltrials.gov/ct2/show/NCT04379271
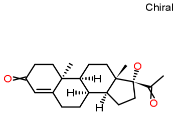	MMV637496	Hydroxyprogesterone	68-96-2	(8S,9S,10S,13S,14S,17R)-17-acetyl-17-hydroxy-10,13-dimethyl-2,6,7,8,9,11,12,14,15,16-decahydro-1H-cyclopenta[a]phenanthren-3-one	C21 H30 O3	330,4611	Launched	Antigonadotropin agent	doi.org/10.1101/2020.03.20.999730
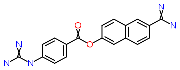	MMV1804176	Nafamostat	81525-10-2	(6-carbamimidoylnaphthalen-2-yl) 4-carbamimidamidobenzoate	C19 H17 N5 O2	347,37058	Launched	Hematologic agent - Anticoagulant	doi.org/10.21203/rs.3.rs-23951/v1

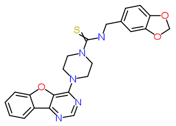	MMV1804173	Amuvatinib	850879-09-3	N-(1,3-benzodioxol-5-ylmethyl)-4-([1]benzofuro[3,2-d]pyrimidin-4-yl)piperazine-1-carbothioamide	$C_{23} H_{21} N_{5} O_{3} S$	447,50954	Ph II, discontinued	Antitumor agent	doi.org/10.21203/rs.3.rs-23951/v1
Chiral 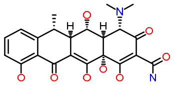	MMV000011	Doxycycline	10592-13-9 564-25-0	(4S,4aR,5S,5aR,6R,12aS)-4-(dimethylamino)-1,5,10,12,12a-pentahydroxy-6-methyl-3,11-dioxo-4a,5,5a,6-tetrahydro-4H-tetracene-2-carboxamide	$C_{22} H_{24} N_{2} O_{8}$	444,43456	Launched	Anti-infective agent - Antibacterial	doi.org/10.1101/2020.03.20.999730
Chiral 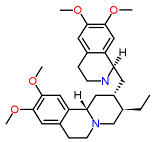	MMV688731	Emetine	316-42-7 483-18-1	(2S,3R,11bS)-2-[[(1R)-6,7-dimethoxy-1,2,3,4-tetrahydroisoquinolin-1-yl]methyl]-3-ethyl-9,10-dimethoxy-2,3,4,6,7,11b-hexahydro-1H-benzo[a]quinolizine	$C_{29} H_{40} N_{2} O_{4}$	480,6389	Launched	Anti-infective agent - Antiparasitic	doi.org/10.21203/rs.3.rs-23951/v1
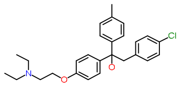	MMV1804244	Triparanol	78-41-1	2-(4-chlorophenyl)-1-[4-[2-(diethylamino)ethoxy]phenyl]-1-(4-methylphenyl)ethanol	$C_{27} H_{32} Cl N O_{2}$	438,00148	Withdrawn	Antilipemic agent	doi.org/10.1101/2020.03.25.008482.
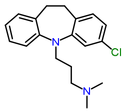	MMV002092	Clomipramine	17321-77-6 303-49-1	3-(2-chloro-5,6-dihydrobenzo[b][1]benzazepin-11-yl)-N,N-dimethylpropan-1-amine	$C_{19} H_{23} Cl N_{2}$	314,859993	Launched	Nervous system agent - Antidepressant	doi.org/10.1101/2020.03.25.008482.
Chiral 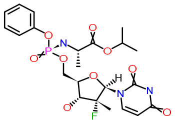	MMV1804189	Sofosbuvir	1190307-88-0	propan-2-yl (2S)-2-[[[(2S,3S,4S,5S)-5-(2,4-dioxopyrimidin-1-yl)-4-fluoro-3-hydroxy-4-methyloxolan-2-yl]methoxy-phenoxyphosphoryl]amino]propanoate	$C_{22} H_{29} F N_{3} O_{9} P$	529,45252	Launched	Anti-infective agent - Antiviral agent	doi.org/10.1016/j.lfs.2020.117592
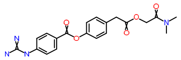	MMV1804242	Camostat	59721-28-7 59721-29-8	[4-[2-[2-(dimethylamino)-2-oxoethoxy]-2-oxoethyl]phenyl] 4-carbamimidamidobenzoate	$C_{20} H_{22} N_{4} O_{5}$	398,41248	Launched	Anti-inflammatory agent - Pancreatitis	doi.org/10.21203/rs.3.rs-23951/v1

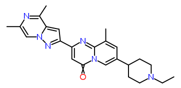	MMV1804249	SMN-C3	1449597-34-5	2-(4,6-dimethylpyrazolo[1,5-a]pyrazin-2-yl)-7-(1-ethylpiperidin-4-yl)-9-methylpyrido[1,2-a]pyrimidin-4-one	C24 H28 N6 O	416,51872	Preclinical	Genetic disorders agent - Spinal muscular atrophy	
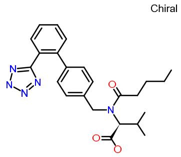	MMV002295	Valsartan	137862-53-4	(2S)-3-methyl-2-[pentanoyl-[[4-[2-(1H-tetrazol-5-yl)phenyl]phenyl]methyl]amino]butanoic acid	C24 H29 N5 O3	435,51876	Launched	Cardiovascular agent - Antihypertensive	doi.org/10.1101/2020.03.20.999730
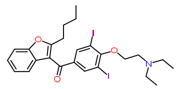	MMV001992	Amiodarone	1951-25-3 19774-82-4	(2-butyl-1-benzofuran-3-yl)-[4-[2-(diethylamino)ethoxy]-3,5-diiodophenyl]methanone	C25 H29 I2 N O3	645,31899	Launched	Cardiovascular agent - Antiarrhythmic	clinicaltrials.gov/ct2/show/NCT04351763
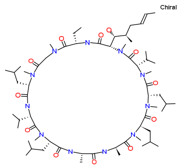	MMV001860	Cyclosporine	59865-13-3	(3S,6S,9R,12R,15S,18S,21S,24S,30S,33S)-30-ethyl-33-[(E,1R,2R)-1-hydroxy-2-methylhex-4-enyl]-1,4,7,10,12,15,19,25,28-nonamethyl-6,9,18,24-tetrakis(2-	C62 H111 N11 O12	1202,61124	Launched	Pharmaceutical immune agent - Immunosuppressant	doi.org/10.1101/2020.03.20.999730
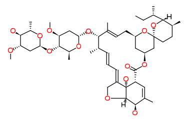	MMV672931	Ivermectin	70288-86-7	rac-(1S,4R,5'R,6S,6'S,8S,10E,12R,13R,14E,16E,20S,21S,24R)-21,24-dihydroxy-5',11,13,22-tetramethyl-6'-[rac-(2R)-butan-2-yl]-12-[rac-(2S,4R,5R,6R)-4-	C48 H74 O14	875,09276	Launched	Anti-infective agent - Antiparasitic	doi.org/10.1016/j.antiviral.2020.104787
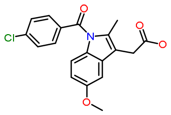	MMV002813	Indomethacin	53-86-1	2-[1-(4-chlorobenzoyl)-5-methoxy-2-methylindol-3-yl]acetic acid	C19 H16 Cl N O4	357,792992	Launched	Anti-inflammatory agent	doi.org/10.1111/ijcp.13535
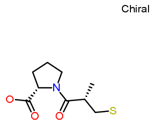	MMV002086	Captopril	62571-86-2	(2S)-1-[(2S)-2-methyl-3-sulfanylpropanoyl]pyrrolidine-2-carboxylic acid	C9 H15 N O3 S	217,2853	Launched	Cardiovascular agent - Antihypertensive	clinicaltrials.gov/ct2/show/NCT04355429

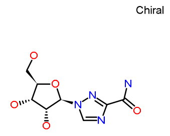	MMV001439	Ribavirin	36791-04-5	1-[(2R,3R,4S,5R)-3,4-dihydroxy-5-(hydroxymethyl)oxolan-2-yl]-1,2,4-triazole-3-carboxamide	C8 H12 N4 O5	244,20468	Launched	Anti-infective agent - Antiviral agent	doi.org/10.1016/S0140-6736(20)31042-4
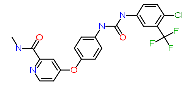	MMV010306	Sorafenib	284461-73-0	4-[4-[[4-chloro-3-(trifluoromethyl)phenyl]carbamoylamino]phenoxy]-N-methylpyridine-2-carboxamide	C21 H16 Cl F3 N4 O3	464,830989	Launched	Antitumor agent - Kidney cancer	doi.org/10.21203/rs.3.rs-23951/v1
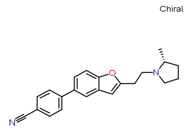	MMV1804225	ABT 239	460746-46-7<br>460748-71-4	4-[2-[2-[(2R)-2-methylpyrrolidin-1-yl]ethyl]-1-benzofuran-5-yl]benzonitrile	C22 H22 N2 O	330,42288	Ph I, discontinued	Nervous system agent	
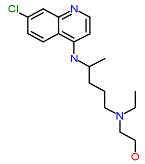	MMV000013	Hydroxychloroquine	118-42-3	2-[4-[(7-chloroquinolin-4-yl)amino]pentyl-ethylamino]ethanol	C18 H26 Cl N3 O	335,87154	Launched	Anti-infective agent - Antimalarial	doi.org/10.1093/cid/ciaa237<br>doi.org/10.4414/smw.2020.20262<br>doi.org/10.1016/j.ijantimicag.2020.105949
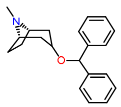	MMV001977	Benztropine	132-17-2<br>86-13-5	rac-(1R,5R)-3-benzhydryloxy-8-methyl-8-azabicyclo[3.2.1]octane	C21 H25 N O	307,4293	Launched	Nervous system agent - Antipsychotic	doi.org/10.1101/2020.03.25.008482.
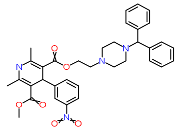	MMV1804194	Manidipine	89226-50-6<br>89226-75-5	2-(4-benzhydrylpiperazin-1-yl)ethyl (5Z)-5-[hydroxy(methoxy)methylidene]-2,6-dimethyl-4-(3-nitrophenyl)-4H-pyridine-3-carboxylate	C35 H38 N4 O6	610,69942	Launched	Cardiovascular agent - Antihypertensive	Corona virus drugs – a brief overview of past, present and future, Journal of PeerScientist 2(2): e1000013
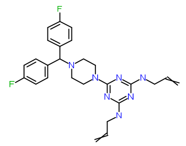	MMV1804175	Almitrine	27469-53-0<br>29608-49-9	6-[4-[bis(4-fluorophenyl)methyl]piperazin-1-yl]-2-N,4-N-bis(prop-2-enyl)-1,3,5-triazine-2,4-diamine	C26 H29 F2 N7	477,55217	Launched	Respiratory system agent	doi.org/10.21203/rs.3.rs-23951/v1

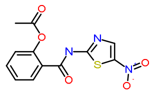	MMV688991	Nitazoxanide	55981-09-4	[2-[(5-nitro-1,3-thiazol-2-yl)carbamoyl]phenyl] acetate	C12 H9 N3 O5 S	307,28196	Launched	Anti-infective agent - Antiparasitic	doi.org/10.20944/preprints202004.0432.v1
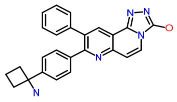	MMV690727	MK-2206	1032349-93-1 1032350-13-2	8-[4-(1-aminocyclobutyl)phenyl]-9-phenyl-[1,2,4]triazolo[3,4-f][1,6]naphthyridin-3-ol	C25 H21 N5 O	407,46713	Ph II, discontinued	Antitumor agent	doi.org/10.1101/2020.04.15.997254
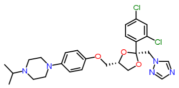	MMV1804243	Terconazole	67915-31-5	1-propan-2-yl-4-[4-[[rac-(2R,4S)-2-(2,4-dichlorophenyl)-2-(1,2,4-triazol-1-ylmethyl)-1,3-dioxolan-4-yl]methoxy]phenyl]piperazine	C26 H31 Cl2 N5 O3	532,471989	Launched	Anti-infective agent - Antifungal	doi.org/10.1016/j.ijantimicag.2020.105969
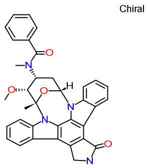	MMV010288	Midostaurin	120685-11-2	N-[(2S,3R,4R,6R)-3-methoxy-2-methyl-16-oxo-29-oxa-1,7,17-triazaoctacyclo[12.12.2.12,6.07,28.08,13.015,19.020,27.021,26]nonacosa-8,10,12,14(28),15(19),2	C35 H30 N4 O4	570,6371	Launched	Antitumor agent - Leukemia	doi.org/10.1101/2020.03.22.002386.
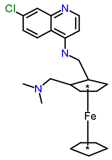	MMV000056	Ferroquine	185055-67-8	7-chloro-N-[[2-[(dimethylamino)methyl]cyclopentyl]methyl]quinolin-4-amine;cyclopentane;iron	C23 H24 Cl Fe N3	433,765992	Ph II	Anti-infective agent - Antimalarial	doi:10.20944/preprints202003.0275.v1
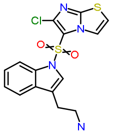	MMV1804248	SAX-187	554403-49-5 1883548-85-3	2-[1-(6-chloroimidazo[2,1-b][1,3]thiazol-5-yl)sulfonylindol-3-yl]ethanamine	C15 H13 Cl N4 O2 S2	380,87232	Ph I, discontinued	Nervous system agent - Antipsychotic	
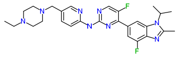	MMV1804174	Abemaciclib	1231929-97-7	N-[5-[(4-ethylpiperazin-1-yl)methyl]pyridin-2-yl]-5-fluoro-4-(7-fluoro-2-methyl-3-propan-2-ylbenzimidazol-5-yl)pyrimidin-2-amine	C27 H32 F2 N8	506,59339	Launched	Antitumor agent - Breast cancer	doi.org/10.1101/2020.03.20.999730

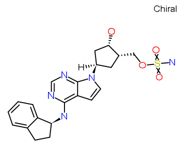	MMV1804191	Pevonedistat	905579-51-3	[(1S,2S,4R)-4-[4-[[(1R)-2,3-dihydro-1H-inden-1-yl]amino]pyrrolo[2,3-d]pyrimidin-7-yl]-2-hydroxycyclopentyl]methyl sulfamate	C21 H25 N5 O4 S	443,5193	Ph III	Antitumor agent	doi.org/10.21203/rs.3.rs-23951/v1 doi.org/10.1038/s41586-020-2286-9
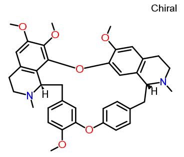	MMV003277	Tetrandrine	518-34-3	(1S,14S)-9,20,21,25-tetramethoxy-15,30-dimethyl-7,23-dioxa-15,30-diazaheptacyclo[22.6.2.23,6.18,12.114,18.027,31.022,33]hexatriaconta-3(36),4,6(35),8(34),9,11,	C38 H42 N2 O6	622,74988	Ph III, discontinued	Antitumor agent	clinicaltrials.gov/ct2/show/NCT04308317
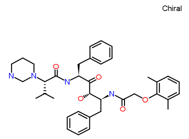	MMV637572	Lopinavir	192725-17-0	(2S)-2-(1,3-diazinan-1-yl)-N-[(2S,4S,5S)-5-[[2-(2,6-dimethylphenoxy)acetyl]amino]-4-hydroxy-3-oxo-1,6-diphenylhexan-2-yl]-3-methylbutanamide	C37 H48 N4 O5	628,80082	Launched	Anti-infective agent - Anti-HIV agent	doi.org/10.1101/2020.03.20.999730
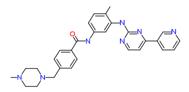	MMV009948	Imatinib	152459-95-5 220127-57-1	4-[(4-methylpiperazin-1-yl)methyl]-N-[4-methyl-3-[(4-pyridin-3-ylpyrimidin-2-yl)amino]phenyl]benzamide	C29 H31 N7 O	493,60274	Launched	Antitumor agent - Leukemia	doi.org/10.1101/2020.03.25.008482.
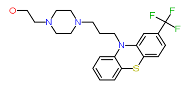	MMV001829	Fluphenazine	146-56-5 69-23-8	2-[4-[3-[2-(trifluoromethyl)phenothiazin-10-yl]propyl]piperazin-1-yl]ethanol	C22 H26 F3 N3 O S	437,52799	Launched	Nervous system agent - Antipsychotic	doi.org/10.1101/2020.03.25.008482.
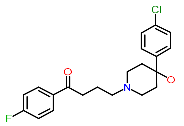	MMV001635	Haloperidol	52-86-8	4-[4-(4-chlorophenyl)-4-hydroxypiperidin-1-yl]-1-(4-fluorophenyl)butan-1-one	C21 H23 Cl F N O2	375,86422	Launched	Nervous system agent - Schizophrenia	doi.org/10.4414/smw.2020.20235
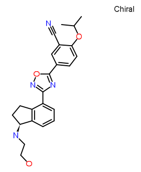	MMV1580492	Ozanimod	1306760-87-1	5-[3-[(1S)-1-(2-hydroxyethylamino)-2,3-dihydro-1H-inden-4-yl]-1,2,4-oxadiazol-5-yl]-2-propan-2-yloxybenzonitrile	C23 H24 N4 O3	404,46166	Launched	Pharmaceutical immune agent - Multiple sclerosis	clinicaltrials.gov/ct2/show/NCT04405102

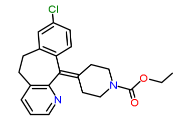	MMV002229	Loratidine	79794-75-5	ethyl 4-(13-chloro-4-azatricyclo[9.4.0.03,8]pentadeca-1(11),3(8),4,6,12,14-hexaen-2-ylidene)piperidine-1-carboxylate	C22 H23 Cl N2 O2	382,88322	Launched	Pharmaceutical immune agent - Antihystaminic	doi.org/10.21203/rs.3.rs-23951/v1
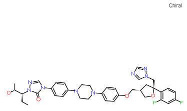	MMV688774	Posaconazole	171228-49-2	4-[4-[4-[4-[[(3R,5R)-5-(2,4-difluorophenyl)-5-(1,2,4-triazol-1-ylmethyl)oxolan-3-yl]methoxy]phenyl]piperazin-1-yl]phenyl]-2-[(2S,3S)-2-hydroxypentan-3-yl]-	C37 H42 F2 N8 O4	700,77739	Launched	Anti-infective agent - Antifungal	doi.org/10.21203/rs.3.rs-23951/v1
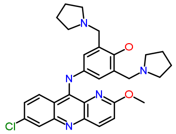	MMV000025	Pyronaridine	74847-35-1 76748-86-2	4-[(7-chloro-2-methoxybenzo[b][1,5]naphthyridin-10-yl)amino]-2,6-bis(pyrrolidin-1-ylmethyl)phenol	C29 H32 Cl N5 O2	518,060989	Launched	Anti-infective agent - Antimalarial	doi.org/10.1101/2020.03.20.999730
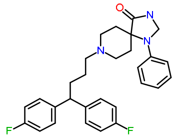	MMV001681	Fluspirilene	1841-19-6	8-[4,4-bis(4-fluorophenyl)butyl]-1-phenyl-1,3,8-triazaspiro[4.5]decan-4-one	C29H31F2N3O	475,582989	Launched	Nervous system agent - Schizophrenia	doi.org/10.1101/2020.03.25.008482.
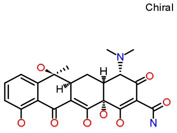	MMV000068	Tetracycline	60-54-8 64-75-5	(4S,4aS,5aS,6S,12aS)-4-(dimethylamino)-1,6,10,12,12a-pentahydroxy-6-methyl-3,11-dioxo-4,4a,5,5a-tetrahydrotetracene-2-carboxamide	C22 H24 N2 O8	444,43455	Launched	Anti-infective agent - Antibiotic	doi.org/10.1016/j.apsb.2020.02.008
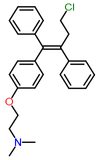	MMV638007	Toremifene	89778-26-7 89778-27-8	2-[4-[(Z)-4-chloro-1,2-diphenylbut-1-enyl]phenoxy]-N,N-dimethylethanamine	C26H28ClNO	405,95962	Launched	Antitumor agent - Breast cancer	doi.org/10.1101/2020.03.25.008482.
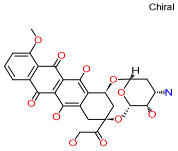	MMV004066	Doxorubicin	23214-92-8 25316-40-9	(7S,9S)-7-[(2R,4S,5S,6S)-4-amino-5-hydroxy-6-methyloxan-2-yl]oxy-6,9,11-trihydroxy-9-(2-hydroxyacetyl)-4-methoxy-8,10-dihydro-7H-tetracene-5,12-dione	C27H29NO11	543,51926	Launched	Anti-infective agent - Antibiotic	doi: 10.1038/s41586-020-2286-9

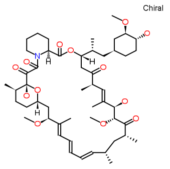	MMV637897	Rapamycin	53123-88-9	(1R,9S,12S,15R,16E,18R,19R,21R,23S,24E,26E,28E,30S,32S,35R)-1,18-dihydroxy-12-[(2R)-1-[(1S,3R,4R)-4-hydroxy-3-methoxycyclohexyl]propan-2-yl]-19,30-dimethoxy-	C51H79NO13	914,17186	Launched	Pharmaceutical immune agent - Immunosuppressant	doi: 10.1038/d41587-020-00013-z
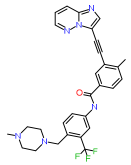	MMV1580167	Ponatinib	943319-70-8	3-(2-imidazo[1,2-b]pyridazin-3-ylethynyl)-4-methyl-N-[4-[(4-methylpiperazin-1-yl)methyl]-3-(trifluoromethyl)phenyl]benzamide	C29 H27 F3 N6 O	532,55949	Launched	Antitumor agent - Leukemia	doi.org/10.1101/2020.02.05.936013
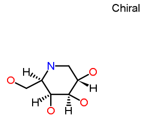	MMV1804188	Migalastat	108147-54-2 75172-81-5	(2R,3S,4R,5S)-2-(hydroxymethyl)piperidine-3,4,5-triol	C6 H13 N O4	163,17172	Launched	Genetic disorders agent - Fabry disease	doi.org/10.1101/2020.03.22.002386
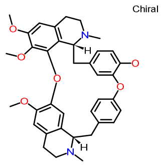	MMV007474	Berbamine	478-61-5	(1S,14R)-20,21,25-trimethoxy-15,30-dimethyl-7,23-dioxa-15,30-diazaheptacyclo[22.6.2.23,6.18,12.114,18.027,31.022,33]hexatriaconta-3,5,8(34),9,11,18(33),19	C37H40N2O6	608,7233	Research	Antitumor agent	doi.org/10.1101/2020.03.20.999730
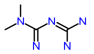	MMV004131	Metformin	1115-70-4 657-24-9	3-(diaminomethylidene)-1,1-dimethylguanidine	C4 H11 N5	129,16364	Launched	Antidiabetic agent	doi.org/10.1016/j.diabres.2020.108183
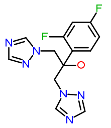	MMV002337	Fluconazole	86386-73-4	2-(2,4-difluorophenyl)-1,3-bis(1,2,4-triazol-1-yl)propan-2-ol	C13H12F2N6O	306,27079	Launched	Anti-infective agent - Antifungal	doi.org/10.1016/j.clim.2020.108413 10.1080/07391102.2020.1763201
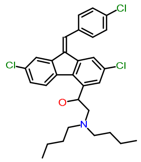	MMV000014	Lumefantrine	82186-77-4	2-(dibutylamino)-1-[(9Z)-2,7-dichloro-9-[(4-chlorophenyl)methylidene]fluoren-4-yl]ethanol	C30 H32 Cl3 N O	528,950988	Launched	Anti-infective agent - Antimalarial	doi.org/10.21203/rs.3.rs-23951/v1

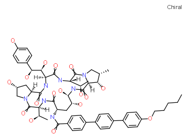	MMV1804247	Anidulafungin	166663-25-8	N-[(3S,6S,9S,11R,15S,18S,20R,21R,24S,25S,26S)-6-[(1S,2S)-1,2-dihydroxy-2-(4-hydroxyphenyl)ethyl]-11,20,21,25-tetrahydroxy-3,15-bis[(1R)-1-hydroxyethyl]-	C58 H73 N7 O17	1140,23692	Launched	Anti-infective agent	doi.org/10.1101/2020.03.20.999730
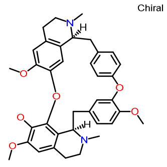	MMV1804250	Hanfangchin B	436-77-1	(1S,14R)-9,20,25-trimethoxy-15,30-dimethyl-7,23-dioxa-15,30-diazaheptacyclo[22.6.2.23,6.18,12.114,18.027,31.022,33]hexatriaconta-3,5,8(34),9,11,18,20,22(	C37 H40 N2 O6	608,7233	Research	Anti-inflammatory agent	
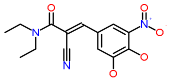	MMV640046	Entacapone	130929-57-6	(E)-2-cyano-3-(3,4-dihydroxy-5-nitrophenyl)-N,N-diethylprop-2-enamide	C14H15N3O5	305,289993	Launched	Nervous system agent - Parkinson's disease	doi.org/10.1038/s41586-020-2286-9
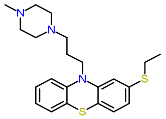	MMV001428	Thiethylperazine	1179-69-7 1420-55-9	2-ethylsulfanyl-10-[3-(4-methylpiperazin-1-yl)propyl]phenothiazine	C22H29N3S2	399,622992	Launched	Gastrointestinal agent - Antivomiting	doi.org/10.1101/2020.03.25.008482.
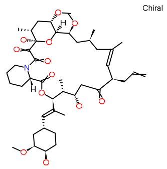	MMV639922	Tacrolimus	104987-11-3	(1R,9S,12S,13R,14S,17R,18E,21S,23S,24R,25S,27R)-1,14-dihydroxy-12-[(E)-1-[(1R,3S,4R)-4-hydroxy-3-methoxycyclohexyl]prop-1-en-2-yl]-23,25-dimethoxy-13,19,21,27-	C44 H69 N O12	804,01816	Launched	Anti-inflammatory agent - Immunosuppresive drug	doi.org/10.1681/ASN.2020030348
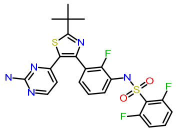	MMV1803334	Dabrafenib	1195765-45-7	N-[3-[5-(2-aminopyrimidin-4-yl)-2-tert-butyl-1,3-thiazol-4-yl]-2-fluorophenyl]-2,6-difluorobenzenesulfonamide	C23 H20 F3 N5 O2 S2	519,56241	Launched	Antitumor agent	
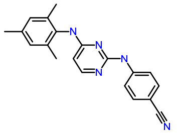	MMV1804311	Dapivirine	244767-67-7	4-[[4-(2,4,6-trimethylanilino)pyrimidin-2-yl]amino]benzonitrile	C20 H19 N5	329,39836	Phase III	Anti-infective agent - Anti-HIV agent	doi.org/10.21203/rs.3.rs-23951/v1

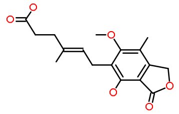	MMV003219	Mycophenolic acid	24280-93-1	(E)-6-(4-hydroxy-6-methoxy-7-methyl-3-oxo-1H-2-benzofuran-5-yl)-4-methylhex-4-enoic acid	C17 H20 O6	320,33709	Launched	Pharmaceutical immune agent - Immunosuppressant	doi.org/10.1101/2020.05.05.079095
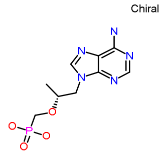	MMV1580478	Tenofovir	147127-20-6	[(2R)-1-(6-aminopurin-9-yl)propan-2-yl]oxymethylphosphonic acid	C9 H14 N5 O4 P	287,21232	Launched	Anti-infective agent - Anti-HIV agent	doi.org/10.1016/j.lfs.2020.117592
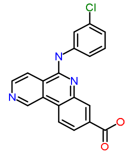	MMV083882	Silmitasertib	1009820-21-6	5-(3-chloroanilino)benzo[c][2,6]naphthyridine-8-carboxylic acid	C19H12ClN3O2	349,776992	Phase I/II	Antitumor agent - Medulloblastoma	doi: 10.1038/d41587-020-00013-z
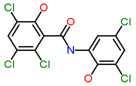	MMV1804313	Oxyclozanide	2277-92-1	2,3,5-trichloro-N-(3,5-dichloro-2-hydroxyphenyl)-6-hydroxybenzamide	C13 H6 Cl5 N O3	401,45664	Research	Anti-infective agent	doi.org/10.1101/2020.03.20.999730
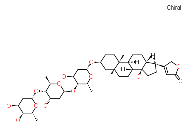	MMV002436	Digitoxin	71-63-6	3-[(3S,5R,8R,9S,10S,13R,14S,17R)-3-[(2R,4S,5S,6R)-5-[(2S,4S,5S,6R)-5-[(2S,4S,5S,6R)-4,5-dihydroxy-6-methyloxan-2-yl]oxy-4-hydroxy-6-methyloxan-	C41 H64 O13	764,93906	Launched	Anti-infective agent	doi.org/10.1101/2020.03.20.999730
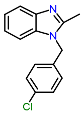	MMV071737	Chlormidazole	3689-76-7 74298-63-8	1-[(4-chlorophenyl)methyl]-2-methylbenzimidazole	C15 H13 Cl N2	256,735994	Launched	Anti-infective agent	doi.org/10.21203/rs.3.rs-23951/v1
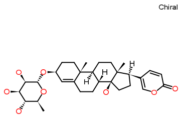	MMV001433	Proscillaridin	466-06-8	5-[(3S,8R,9S,10R,13R,14S,17R)-14-hydroxy-10,13-dimethyl-3-[(2R,3R,4R,5R,6S)-3,4,5-trihydroxy-6-methyloxan-2-yl]oxy-1,2,3,6,7,8,9,11,12,15,1	C30 H42 O8	530,64968	Research	Cardiovascular agent	doi.org/10.1101/2020.03.20.999730

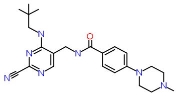	MMV1804353	Dutacatib	501000-36-8	N-[[2-cyano-4-(2,2-dimethylpropylamino)pyrimidin-5-yl]methyl]-4-(4-methylpiperazin-1-yl)benzamide	C23 H31 N7 O	421,53854	Research	Antitumor agent	
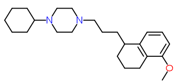	MMV1804354	PB 28	172906-90-0 172907-03-8	1-cyclohexyl-4-[3-(5-methoxy-1,2,3,4-tetrahydronaphthalen-1-yl)propyl]piperazine	C24 H38 N2 O	370,57132	Research	Antitumor agent	doi.org/10.1038/s41586-020-2286-9
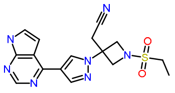	MMV690755	Baricitinib	1187594-09-7	2-[1-ethylsulfonyl-3-[4-(7H-pyrrolo[2,3-d]pyrimidin-4-yl)pyrazol-1-yl]azetidin-3-yl]acetonitrile	C16 H17 N7 O2 S	371,41687	Launched	Anti-inflammatory agent	doi: 10.1038/d41587-020-00013-z
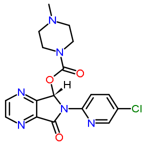	MMV1804617	Eszopiclone	138729-47-2	[(7S)-6-(5-chloropyridin-2-yl)-5-oxo-7H-pyrrolo[3,4-b]pyrazin-7-yl] 4-methylpiperazine-1-carboxylate	C17 H17 Cl N6 O3	388,80828	Launched	Nervous system agent - Insomnia	doi.org/10.1002/2211-5463.12875
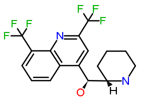	MMV000016	(+)-Mefloquine	51742-86-0 51742-87-1	(S)-[2,8-bis(trifluoromethyl)quinolin-4-yl]-[(2R)-piperidin-2-yl]methanol	C17H16F6N2O	378,31216	Launched	Anti-infective agent - Antimalarial	doi.org/10.1101/2020.03.20.999730
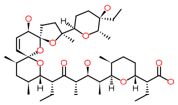	MMV1804312	Salinomycin	53003-10-4 55721-31-8	(2R)-2-[(2R,5S,6R)-6-[(2S,3S,4S,6R)-6-[(3S,5S,7R,9S,10S,12R,15R)-3-[(2R,5R,6S)-5-ethyl-5-hydroxy-6-methyloxan-2-yl]-15-hydroxy-3,10,12-trimethyl-4,6,8-	C42 H70 O11	750,9986	Research	Anti-infective agent - Anti microbial agent	doi.org/10.1101/2020.03.20.999730
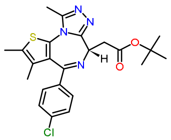	MMV1804357	JQ 1	1268524-70-4	tert-butyl 2-[(9S)-7-(4-chlorophenyl)-4,5,13-trimethyl-3-thia-1,8,11,12-tetrazatricyclo[8.3.0.02,6]trideca-2(6),4,7,10,12-pentaen-9-yl]acetate	C23 H25 Cl N4 O2 S	456,9882	Research	Antitumor agent	doi: 10.1007/s10930-020-09901-4

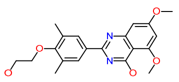	MMV1804356	RVX 208	1044870-39-4	2-[4-(2-hydroxyethoxy)-3,5-dimethylphenyl]-5,7-dimethoxyquinazolin-4-ol	C20 H22 N2 O5	370,39908	Phase III	Cardiovascular agent	doi: 10.1007/s10930-020-09901-4
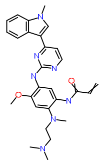	MMV690733	Osimertinib	1421373-65-0 1421373-66-1	N-[2-[2-(dimethylamino)ethyl-methylamino]-4-methoxy-5-[[4-(1-methylindol-3-yl)pyrimidin-2-yl]amino]phenyl]prop-2-enamide	C28 H33 N7 O2	499,60732	Launched	Antitumor agent	doi.org/10.1016/j.ejca.2020.04.004
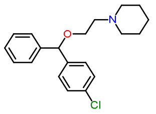	MMV002291	Cloperastine	14984-68-0 3703-76-2	1-[2-[(4-chlorophenyl)-phenylmethoxy]ethyl]piperidine	C20H24ClNO	329,870993	Launched	Pharmaceutical immune agent	doi.org/10.1038/s41586-020-2286-9
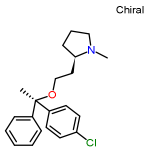	MMV637229	Clemastine	14976-57-9 15686-51-8	(2R)-2-[2-[(1R)-1-(4-chlorophenyl)-1-phenylethoxy]ethyl]-1-methylpyrrolidine	C21H26ClNO	343,89024	Launched	Pharmaceutical immune agent	doi.org/10.1038/s41586-020-2286-9
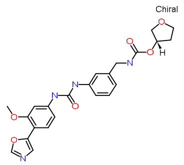	MMV1804359	Merimepodib	198821-22-6	[(3S)-oxolan-3-yl] N-[[3-[[3-methoxy-4-(1,3-oxazol-5-yl)phenyl]carbamoylamino]phenyl]methyl]carbamate	C23 H24 N4 O6	452,45986	Phase IIb	Anti-infective agent - Antiviral agent	doi.org/10.1038/s41586-020-2286-9
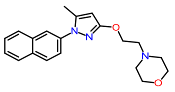	MMV1804360	E 52862	1265917-14-3 878141-96-9	4-[2-(5-methyl-1-naphthalen-2-ylpyrazol-3-yl)oxyethyl]morpholine	C20 H23 N3 O2	337,41552	Phase II	Nervous system agent - Neuropathic pain	doi: 10.1007/s10930-020-09901-4
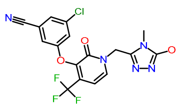	MMV1804620	Doravirine	1338225-97-0	3-chloro-5-[1-[(5-hydroxy-4-methyl-1,2,4-triazol-3-yl)methyl]-2-oxo-4-(trifluoromethyl)pyridin-3-yl]oxybenzonitrile	C17 H11 Cl F3 N5 O3	425,74915	Launched	Anti-infective agent - Anti-HIV agent	doi.org/10.26434/chemrxiv.12101457.v1

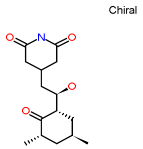	MMV000031	Cycloheximide	66-81-9	4-[(2R)-2-[(1S,3S,5S)-3,5-dimethyl-2-oxocyclohexyl]-2-hydroxyethyl]piperidine-2,6-dione	C15H23NO4	281,34742	Research	Agricultural agent - Fungicid	doi.org/10.21203/rs.3.rs-23951/v1
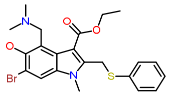	MMV1804355	Umifenovir	131707-25-0	ethyl 6-bromo-4-[(dimethylamino)methyl]-5-hydroxy-1-methyl-2-(phenylsulfanylmethyl)indole-3-carboxylate	C22 H25 Br N2 O3 S	477,4145	Launched	Anti-infective agent - Antiviral agent	doi: 10.1038/d41587-020-00013-z
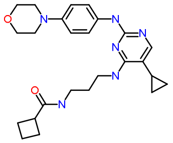	MMV1804409	MRT 68601		N-[3-[[5-cyclopropyl-2-(4-morpholin-4-ylanilino)pyrimidin-4-yl]amino]propyl]cyclobutanecarboxamide	C25 H34 N6 O2	450,57646	Research	Antitumor agent	doi.org/10.1038/s41586-020-2286-9
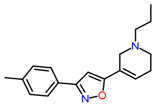	MMV1804408	PD 144418	154130-99-1 1794760-28-3	3-(4-methylphenyl)-5-(1-propyl-3,6-dihydro-2H-pyridin-5-yl)-1,2-oxazole	C18 H22 N2 O	282,38008	Research	Nervous system agent	doi.org/10.1038/s41586-020-2286-9
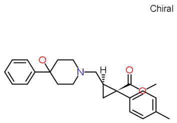	MMV1804411	(RS)-PPCC	932736-91-9 932736-90-8	methyl (1S,2R)-2-[(4-hydroxy-4-phenylpiperidin-1-yl)methyl]-1-(4-methylphenyl)cyclopropane-1-carboxylate	C24 H29 N O3	379,49196	Research	Anti-infective agent - Anti-HIV agent	doi.org/10.1038/s41586-020-2286-9
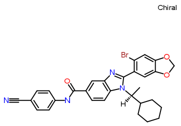	MMV1804479	AZ3451	2100284-59-9	2-(6-bromo-1,3-benzodioxol-5-yl)-N-(4-cyanophenyl)-1-[(1S)-1-cyclohexylethyl]benzimidazole-5-carboxamide	C30 H27 Br N4 O3	571,46438	Research	Anti-inflammatory agent	doi: 10.1007/s10930-020-09901-4
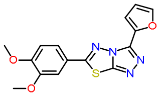	MMV1804484	ZINC4326719	799786-73-5	6-(3,4-dimethoxyphenyl)-3-(furan-2-yl)-[1,2,4]triazolo[3,4-b][1,3,4]thiadiazole	C15 H12 N4 O3 S	328,34578	Research	Antitumor agent	doi: 10.1007/s10930-020-09901-4

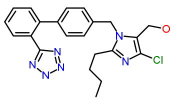	MMV002230	Losartan	114798-26-4	[2-butyl-5-chloro-3-[[4-[2-(2H-tetrazol-5-yl)phenyl]phenyl]methyl]imidazol-4-yl]methanol	C22H23ClN6O	422,919992	Launched	Cardiovascular agent - Antihypertensive	doi.org/10.1016/S2213-2600(20)30153-3
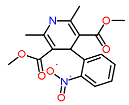	MMV637687	Nifedipine	21829-25-4	dimethyl 2,6-dimethyl-4-(2-nitrophenyl)-1,4-dihydropyridine-3,5-dicarboxylate	C17H18N2O6	346,33462	Launched	Cardiovascular agent - Antihypertensive	doi.org/10.7759/cureus.8069
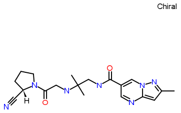	MMV1804621	Anagliptin	739366-20-2	N-[2-[[2-[(2S)-2-cyanopyrrolidin-1-yl]-2-oxoethyl]amino]-2-methylpropyl]-2-methylpyrazolo[1,5-a]pyrimidine-6-carboxamide	C19 H25 N7 O2	383,4475	Launched	Antidiabetic agent	doi.org/10.3390/molecules25112529
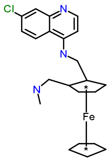	MMV892669	Desmethyl ferroquine	903546-18-9	7-chloro-N-[[2-(methylaminomethyl)cyclopentyl]methyl]quinolin-4-amine;cyclopentane;iron	C22 H22 Cl Fe N3	419,72818	Research	Anti-infective agent - Antimalarial	
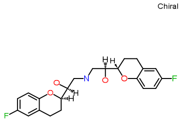	MMV1804412	Nebivolol	152520-56-4 118457-14-0	(1S)-1-[(2R)-6-fluoro-3,4-dihydro-2H-chromen-2-yl]-2-[[(2S)-2-[(2S)-6-fluoro-3,4-dihydro-2H-chromen-2-yl]-2-hydroxyethyl]amino]ethanol	C22 H25 F2 N O4	405,43501	Launched	Cardiovascular agent - Antihypertensive	doi.org/10.1159/000507914
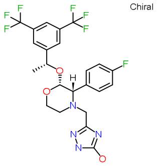	MMV1804480	Aprepitant	170729-80-3	3-[[(2R,3S)-2-[(1R)-1-[3,5-bis(trifluoromethyl)phenyl]ethoxy]-3-(4-fluorophenyl)morpholin-4-yl]methyl]-1H-1,2,4-triazol-5-ol	C23 H21 F7 N4 O3	534,42666	Launched	Gastrointestinal agent	doi.org/10.3390/ijerph17072323
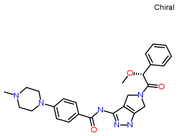	MMV676600	Danusertib	827318-97-8	N-[5-[(2R)-2-methoxy-2-phenylacetyl]-4,6-dihydro-1H-pyrrolo[3,4-c]pyrazol-3-yl]-4-(4-methylpiperazin-1-yl)benzamide	C26 H30 N6 O3	474,5548	Phase II	Antitumor agent - Multiple Myeloma	doi.org/10.3390/ph13060132

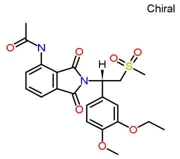	MMV1804482	Apremilast	608141-41-9	N-[2-[(1S)-1-(3-ethoxy-4-methoxyphenyl)-2-methylsulfonylethyl]-1,3-dioxoisoindol-4-yl]acetamide	C22 H24 N2 O7 S	460,50016	Launched	Anti-inflammatory agent - Psoriasis	doi.org/10.1111/dth.13668
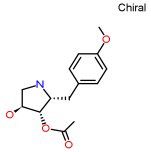	MMV1634116	(-)-Anisomycin	22862-76-6	[(2R,3S,4S)-4-hydroxy-2-[(4-methoxyphenyl)methyl] pyrrolidin-3-yl] acetate	C14H19NO4	265,30496	Launched	Anti-infective agent - Antibiotic	doi.org/10.1101/2020.03.25.008482
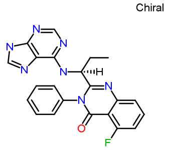	MMV1803325	Idelalisib	870281-82-6	5-fluoro-3-phenyl-2-[(1S)-1-(9H-purin-6-ylamino)propyl]quinazolin-4-one	C22 H18 F N7 O	415,42302	Launched	Antitumor agent - Blood cancer	doi.org/10.26434/chemrxiv.12155523.v1
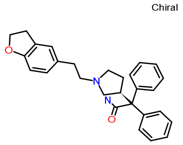	MMV1580132	Darifenacin	133099-07-7	2-[(3S)-1-[2-(2,3-dihydro-1-benzofuran-5-yl)ethyl]pyrrolidin-3-yl]-2,2-diphenylacetamide	C28 H30 N2 O2	426,55	Launched	Nervous system agent	doi.org/10.26434/chemrxiv.12430919.v1
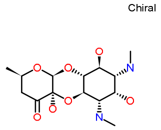	MMV002649	Spectinomycin	1695-77-8<br>22189-32-8	(1R,3S,5R,8R,10R,11S,12S,13R,14S)-8,12,14-trihydroxy-5-methyl-11,13-bis(methylamino)-2,4,9-trioxatricyclo[8.4.0.03,8]tetradecan-7-one	C14H24N2O7	332,34956	Launched	Anti-infective agent - Antibiotic	
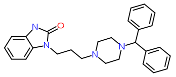	MMV003406	Oxatomide	60607-34-3<br>65215-19-2	3-[3-(4-benzhydrylpiperazin-1-yl)propyl]-1H-benzimidazol-2-one	C27H30N4O	426,5533	Launched	Pharmaceutical immune agent - Antihystaminic	doi.org/10.26434/chemrxiv.12227363.v1
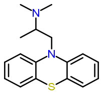	MMV003186	Promethazine	58-33-3<br>60-87-7	N,N-dimethyl-1-phenothiazin-10-ylpropan-2-amine	C17H20N2S	284,424994	Launched	Pharmaceutical immune agent - Antihystaminic	doi.org/10.1101/2020.03.25.008482.

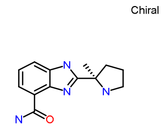	MMV690561	Veliparib	912444-00-9	2-[(2R)-2-methylpyrrolidin-2-yl]-1H-benzimidazole-4-carboxamide	C13 H16 N4 O	244,29234	Phase III	Antitumor agent	doi.org/10.1111/bph.15137
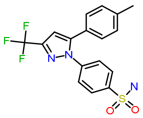	MMV002641	Celecoxib	169590-42-5	4-[5-(4-methylphenyl)-3-(trifluoromethyl)pyrazol-1-yl]benzenesulfonamide	C17H14F3N3O2S	381,375991	Launched	Anti-inflammatory agent	doi.org/10.2139/ssrn.3570102
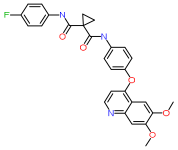	MMV1804483	Cabozantinib	849217-68-1	1-N-[4-(6,7-dimethoxyquinolin-4-yl)oxyphenyl]-1-N'-(4-fluorophenyl)cyclopropane-1,1-dicarboxamide	C28 H24 F N3 O5	501,50566	Launched	Antitumor agent - Thyroid cancer	
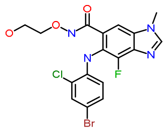	MMV639974	Selumetinib	606143-52-6	6-(4-bromo-2-chloroanilino)-7-fluoro-N-(2-hydroxyethoxy)-3-methylbenzimidazole-5-carboxamide	C17H15BrClFN4O3	457,691988	Launched	Antitumor agent - Neurofibromatosis	doi.org/10.26434/chemrxiv.12148764.v1
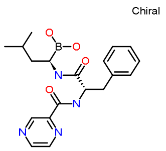	MMV009415	Bortezomib	179324-69-7	[(1R)-3-methyl-1-[[(2S)-3-phenyl-2-(pyrazine-2-carbonylamino)propanoyl]amino]butyl]boronic acid	C19H25BN4O4	384,2372	Launched	Antitumor agent - Multiple myeloma	doi.org/10.3390/ijms21103622
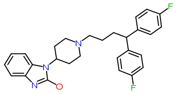	MMV002137	Pimozide	2062-78-4	1-[1-[4,4-bis(4-fluorophenyl)butyl]piperidin-4-yl]benzimidazol-2-ol	C28 H29 F2 N3 O	461,54617	Launched	Nervous system agent - Antipsychotic	doi.org/10.1101/2020.05.23.112235
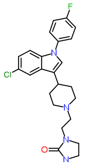	MMV1804478	Sertindole	106516-24-9	1-[2-[4-[5-chloro-1-(4-fluorophenyl)indol-3-yl]piperidin-1-yl]ethyl]imidazolidin-2-one	C24 H26 Cl F N4 O	440,94084	Launched	Nervous system agent - Antipsychotic	doi.org/10.1101/2020.02.05.936013

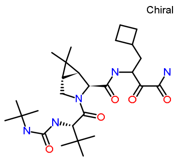	MMV1580497	Boceprevir	394730-60-0	(1R,2S,5S)-N-(4-amino-1-cyclobutyl-3,4-dioxobutan-2-yl)-3-[(2S)-2-(tert-butylcarbamoylamino)-3,3-dimethylbutanoyl]-6,6-dimethyl-3-azabicyclo[3.1.0]hexane-	C27 H45 N5 O5	519,6767	Launched	Anti-infective agent - Antiviral agent	doi.org/10.1038/s41422-020-0356-z
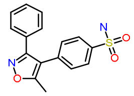	MMV002260	Valdecoxib	181695-72-7	4-(5-methyl-3-phenyl-1,2-oxazol-4-yl)benzenesulfonamide	C16H14N2O3S	314,362993	Launched	Anti-inflammatory agent - Osteoarthritis, Rheumatoid arthritis	doi.org/10.3390/ijms21113793
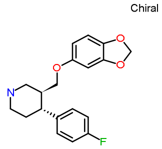	MMV001885	Paroxetine	61869-08-7	(3S,4R)-3-(1,3-benzodioxol-5-yloxymethyl)-4-(4-fluorophenyl)piperidine	C19H20FNO3	329,3654	Launched	Nervous system agent - Antidepressant	doi.org/10.1038/s41421-020-0153-3
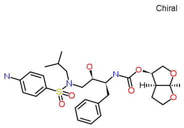	MMV640037	Darunavir	206361-99-1 635728-49-3	[(3aS,4R,6aR)-2,3,3a,4,5,6a-hexahydrofuro[2,3-b]furan-4-yl] N-[(2S,3R)-4-[(4-aminophenyl)sulfonyl-(2-methylpropyl)amino]-3-hydroxy-1-	C27 H37 N3 O7 S	547,66358	Launched	Anti-infective agent - Anti-HIV agent	doi.org/10.2174/0929867327666200416131117
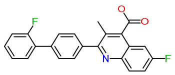	MMV1804517	Brequinar	96187-53-0	6-fluoro-2-[4-(2-fluorophenyl)phenyl]-3-methylquinoline-4-carboxylic acid	C23 H15 F2 N O2	375,36751	Ph III, discontinued	Antitumor agent	doi.org/10.1016/j.bj.2020.05.001
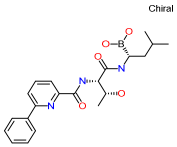	MMV690548	Delanzomib	847499-27-8	[(1R)-1-[[(2S,3R)-3-hydroxy-2-[(6-phenylpyridine-2-carbonyl)amino]butanoyl]amino]-3-methylbutyl]boronic acid	C21 H28 B N3 O5	413,27512	Phase I/II	Antitumor agent - Multiple Myeloma	doi.org/10.26434/chemrxiv.12047346.v1
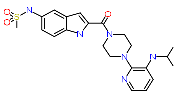	MMV637283	Delavirdine	136817-59-9 147221-93-0	N-[2-[4-[3-(propan-2-ylamino)pyridin-2-yl]piperazine-1-carbonyl]-1H-indol-5-yl]methanesulfonamide	C22H28N6O3S	456,568992	Launched	Anti-infective agent - Anti-HIV agent	doi.org/10.26434/chemrxiv.12210845.v1

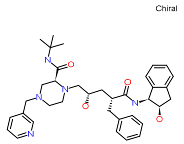	MMV659198	Indinavir	150378-17-9 157810-81-6	(2S)-1-[(2S,4R)-4-benzyl-2-hydroxy-5-[[(1S,2R)-2-hydroxy-2,3-dihydro-1H-inden-1-yl]amino]-5-oxopentyl]-N-tert-butyl-4-(pyridin-3-ylmethyl)piperazine-2-carboxamide	C36 H47 N5 O4	613,78948	Launched	Anti-infective agent - Anti-HIV agent	doi.org/10.1039/d0ra01899f
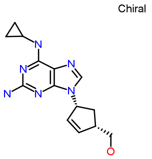	MMV636937	Abacavir	136470-78-5	[(1S,4R)-4-[2-amino-6-(cyclopropylamino)purin-9-yl]cyclopent-2-en-1-yl]methanol	C14H18N6O	286,33232	Launched	Anti-infective agent - Anti-HIV agent	doi.org/10.1016/S2352-3018(20)30111-9
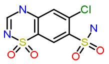	MMV001901	Chlorothiazide	58-94-6	6-chloro-1,1-dioxo-4H-1?6,2,4-benzothiadiazine-7-sulfonamide	C7H6ClN3O4S2	295,722994	Launched	Cardiovascular agent - Antihypertensive	doi.org/10.1007/s002329900314
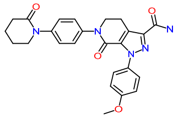	MMV1803326	Apixaban	503612-47-3	1-(4-methoxyphenyl)-7-oxo-6-[4-(2-oxopiperidin-1-yl)phenyl]-4,5-dihydropyrazolo[3,4-c]pyridine-3-carboxamide	C25 H25 N5 O4	459,4971	Launched	Hematologic agent - Anticoagulant	doi.org/10.1007/s11739-020-02331-1
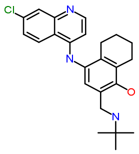	MMV000017	Naphtoquine	173531-57-2 173531-58-3	2-[(tert-butylamino)methyl]-4-[(7-chloroquinolin-4-yl)amino]-5,6,7,8-tetrahydronaphthalen-1-ol	C24 H28 Cl N3 O	409,95161	Research	Anti-infective agent - Antimalarial	10.26434/chemrxiv.12136002
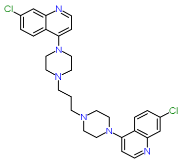	MMV000022	Piperaquine	4085-31-8 915967-82-7	7-chloro-4-[4-[3-[4-(7-chloroquinolin-4-yl)piperazin-1-yl]propyl]piperazin-1-yl]quinoline	C29 H32 Cl2 N6	535,522989	Launched	Anti-infective agent - Antimalarial	doi.org/10.1016/j.drup.2020.100719 doi.org/10.1016/j.imu.2020.100345
