# Supplementary material for: Medicines for Malaria Venture COVID Box: a source for repurposing drugs with antifungal activity against human pathogenic fungi
Source: Mem Inst Oswaldo Cruz. 2021 Nov 8;116:e210207. doi: 10.1590/0074-02760210207 (PMC8577065; doi:10.1590/0074-02760210207)
Supplement: Supplementary file 2 [file 1678-8060-mioc-116-e210207-s2.pdf]

REFERENCES

1. Anséhn S, Granström S, Höjer H, Nilsson L, Akesson E, Lundin A, et al. *In-vitro* effects of *Candida albicans* of amphotericin B combined with other antibiotics. Preliminary observations. Scand J Infect Dis. 1976; 9: 62-6.

2. Antoszczak M, Huczyński A. Salinomycin and its derivatives - A new class of multiple-targeted "magic bullets." Eur J Med Chem. 2019; 176: 208-27.

3. Bastidas RJ, Shertz CA, Lee SC, Heitman J, Cardenas ME. Rapamycin exerts antifungal activity *in vitro* and *in vivo* against *Mucor circinelloides* via FKBP12-dependent inhibition of Tor. Eukaryot Cell. 2012; 11: 270-81.

4. Blankenship JR, Singh N, Alexander BD, Heitman J. *Cryptococcus neoformans* isolates from transplant recipients are not selected for resistance to calcineurin inhibitors by current immunosuppressive regimens. J Clin Microbiol. 2005; 43: 464-7.

5. Borba-Santos LP, Reis de Sá LF, Ramos JA, Rodrigues AM, Camargo ZP de, Rozental S, et al. Tacrolimus increases the effectiveness of itraconazole and fluconazole against *Sporothrix* spp. Front Microbiol. 2017; 8: 1759.

6. Chen SC-A, Lewis RE, Kontoyiannis DP. Direct effects of non-antifungal agents used in cancer chemotherapy and organ transplantation on the development and virulence of *Candida* and *Aspergillus* species. Virulence. 2011; 2: 280-95.

7. Cordeiro RA, Macedo RB, Teixeira CEC, Marques FJF, Bandeira TJPG, Moreira JLB, et al. The calcineurin inhibitor cyclosporin A exhibits synergism with antifungals against *Candida parapsilosis* species complex. J Med Microbiol. 2014; 63: 936-44.

8. Cruz MC, Del Poeta M, Wang P, Wenger R, Zenke G, Quesniaux VF, et al. Immunosuppressive and nonimmunosuppressive cyclosporine analogs are toxic to the opportunistic fungal pathogen *Cryptococcus neoformans* via cyclophilin-dependent inhibition of calcineurin. Antimicrob Agents Chemother. 2000; 44: 143-9.

9. Cruz MC, Goldstein AL, Blankenship J, Del Poeta M, Perfect JR, McCusker JH, et al. Rapamycin and less immunosuppressive analogs are toxic to *Candida albicans* and *Cryptococcus neoformans* via FKBP12-dependent inhibition of TOR. Antimicrob Agents Chemother. 2001; 45: 3162-70.

10. Dehdashti SJ, Abbott J, Nguyen D-T, McKew JC, Williamson PR, Zheng W. A high-throughput screening assay for assessing the viability of *Cryptococcus neoformans* under nutrient starvation conditions. Anal Bioanal Chem. 2013; 405: 6823-9.

11. Del Poeta M, Cruz MC, Cardenas ME, Perfect JR, Heitman J. Synergistic antifungal activities of bafilomycin A(1), fluconazole, and the pneumocandin MK-0991/caspofungin acetate (L-743,873) with calcineurin inhibitors FK506 and L-685,818 against *Cryptococcus neoformans*. Antimicrob Agents Chemother. 2000; 44: 739-46.

12. El-Azizi M. Enhancement of the in vitro activity of amphotericin B against the biofilms of non-*albicans Candida* spp. by rifampicin and doxycycline. J Med Microbiol. 2007; 56: 645-9.

13. Fiori A, Van Dijck P. Potent synergistic effect of doxycycline with fluconazole against *Candida albicans* is mediated by interference with iron homeostasis. Antimicrob Agents Chemother. 2012; 56: 3785-96.

14. Gao Y, Li H, Liu S, Zhang X, Sun S. Synergistic effect of fluconazole and doxycycline against *Candida albicans* biofilms resulting from calcium fluctuation and downregulation of fluconazole-inducible efflux pump gene overexpression. J Med Microbiol. 2014; 63: 956-61.

15. Gao L, Sun Y. *In vitro* interactions of antifungal agents and tacrolimus against *Aspergillus* biofilms. Antimicrob Agents Chemother. 2015; 59: 7097-9.

16. Gao L, Sun Y, He C, Zeng T, Li M. Synergistic effects of tacrolimus and azoles against *Exophiala dermatitidis*. Antimicrob Agents Chemother. 2017; 61: e00948-17.

17. Gao Y, Zhang C, Lu C, Liu P, Li Y, Li H, et al. Synergistic effect of doxycycline and fluconazole against *Candida albicans* biofilms and the impact of calcium channel blockers. FEMS Yeast Res. 2013; 13: 453-62.

18. Garcia C, Burgain A, Chaillot J, Pic É, Khemiri I, Sellam A. A phenotypic small-molecule screen identifies halogenated salicylanilides as inhibitors of fungal morphogenesis, biofilm formation and host cell invasion. Sci Rep. 2018; 8: 11559.

19. Gu W, Yu Q, Yu C, Sun S. *In vivo* activity of fluconazole/tetracycline combinations in *Galleria mellonella* with resistant *Candida albicans* infection. J Glob Antimicrob Resist. 2018; 13: 74-80.

20. Hooper RW, Ashcraft DS, Pankey GA. *In vitro* synergy with fluconazole plus doxycycline or tigecycline against clinical *Candida glabrata* isolates. Med Mycol. 2019; 57: 122-6.

21. Hughes CE, Harris C, Peterson LR, Gerding DN. Enhancement of the *in vitro* activity of amphotericin B against *Aspergillus* spp. by tetracycline analogs. Antimicrob Agents Chemother. 1984; 26: 837-40.

22. Jia W, Zhang H, Li C, Li G, Liu X, Wei J. The calcineruin inhibitor cyclosporine a synergistically enhances the susceptibility of *Candida albicans* biofilms to fluconazole by multiple mechanisms. BMC Microbiol. 2016; 16: 113.

23. Kirkland TN, Fierer J. Cyclosporin A inhibits *Coccidioides immitis in vitro* and *in vivo*. Antimicrob Agents Chemother. 1983; 24: 921-4.

24. Kontoyiannis DP, Lewis RE, Osherov N, Albert ND, May GS. Combination of caspofungin with inhibitors of the calcineurin pathway attenuates growth *in vitro* in *Aspergillus* species. J Antimicrob Chemother. 2003; 51: 313-6.

25. Krajewska-Kułak E, Niczyporuk W. Effects of cyclosporin A on mycelial transformation of *Candida albicans* cells in human serum. J Eur Acad Dermatol Venereol. 1998; 10: 191-2.

26. Krajewska-Kułak E, Niczyporuk W, Szynaka B. Ultrastructural changes of *Candida albicans* under influence of cyclosporin A. Rocz Akad Med Bialymst. 1997; 42(Suppl. 2): 208-11.

27. Kubiça TF, Denardi LB, Azevedo MI, Oliveira V, Severo LC, Santurio JM, et al. Antifungal activities of tacrolimus in combination with antifungal agents against fluconazole-susceptible and fluconazole-resistant *Trichosporon asahii* isolates. Braz J Infect Dis. 2016; 20: 539-45.

28. Kunz J, Hall MN. Cyclosporin A, FK506 and rapamycin: more than just immunosuppression. Trends Biochem Sci. 1993; 18: 334-8.

29. Lew MA, Beckett KM, Levin MJ. Antifungal activity of four tetracycline analogues against *Candida albicans in vitro*: potentiation by amphotericin B. J Infect Dis. 1977; 136: 263-70.

30. Lewis RE, Ben-Ami R, Best L, Albert N, Walsh TJ, Kontoyiannis DP. Tacrolimus enhances the potency of posaconazole against *Rhizopus oryzae in vitro* and in an experimental model of mucormycosis. J Infect Dis. 2013; 207: 834-41.

31. Li Y, Sun S, Guo Q, Ma L, Shi C, Su L, et al. *In vitro* interaction between azoles and cyclosporin A against clinical isolates of *Candida albicans* determined by the chequerboard method and time-kill curves. J Antimicrob Chemother. 2008; 61: 577-85.

32. Lin C-J, Chang Y-L, Yang Y-L, Chen Y-L. Natural alkaloid tryptanthrin exhibits novel anticryptococcal activity. Med Mycol. 2020; doi: 10.1093/mmy/myaa074.

33. Lown L, Peters BM, Walraven CJ, Noverr MC, Lee SA. An optimized lock solution containing micafungin, ethanol and doxycycline inhibits *Candida albicans* and mixed *C. albicans - Staphyloccoccus aureus* biofilms. PLoS One. 2016; 11: e0159225.

34. Marchetti O, Entenza JM, Sanglard D, Bille J, Glauser MP, Moreillon P. Fluconazole plus cyclosporine: a fungicidal combination effective against experimental endocarditis due to *Candida albicans*. Antimicrob Agents Chemother. 2000; 44: 2932-8.

35. Marchetti O, Moreillon P, Entenza JM, Vouillamoz J, Glauser MP, Bille J, et al. Fungicidal synergism of fluconazole and cyclosporine in *Candida albicans* is not dependent on multidrug efflux transporters encoded by the *CDR1*, *CDR2*, *CaMDR1*, and *FLU1* genes. Antimicrob Agents Chemother. 2003; 47: 1565-70.

36. Marchetti O, Moreillon P, Glauser MP, Bille J, Sanglard D. Potent synergism of the combination of fluconazole and cyclosporine in *Candida albicans*. Antimicrob Agents Chemother. 2000; 44: 2373-81.

37. Massuda TYC, Nagashima LA, Leonello PC, Kaminami MS, Mantovani MS, Sano A, et al. Cyclosporin A treatment and decreased fungal load/antigenemia in experimental murine paracoccidioidomycosis. Mycopathologia. 2011; 171: 161-9.

38. Miceli MH, Bernardo SM, Lee SA. *In vitro* analyses of the combination of high-dose doxycycline and antifungal agents against *Candida albicans* biofilms. Int J Antimicrob Agents. 2009; 34: 326-32.

39. Miyazaki Y, Shibuya M, Sugawara H, Kawaguchi O, Hirsoe C. Salinomycin, a new polyether antibiotic. J Antibiot (Tokyo). 1974; 27: 814-21.

40. Mody CH, Toews GB, Lipscomb MF. Cyclosporin A inhibits the growth of *Cryptococcus neoformans* in a murine model. Infect Immun. 1988; 56: 7-12.

41. Narreddy S, Manavathu E, Chandrasekar PH, Alangaden GJ, Revankar SG. *In vitro* interaction of posaconazole with calcineurin inhibitors and sirolimus against zygomycetes. J Antimicrob Chemother. 2010; 65: 701-3.

42. Odom A, Del Poeta M, Perfect J, Heitman J. The immunosuppressant FK506 and its nonimmunosuppressive analog L-685,818 are toxic to *Cryptococcus neoformans* by inhibition of a common target protein. Antimicrob Agents Chemother. 1997; 41: 156-61.

43. Ogita A, Konishi Y, Borjihan B, Fujita K, Tanaka T. Synergistic fungicidal activities of polymyxin B and ionophores, and their dependence on direct disruptive action of polymyxin B on fungal vacuole. J Antibiot (Tokyo). 2009; 62: 81-7.

44. Onyewu C, Blankenship JR, Del Poeta M, Heitman J. Ergosterol biosynthesis inhibitors become fungicidal when combined with calcineurin inhibitors against *Candida albicans*, *Candida glabrata*, and *Candida krusei*. Antimicrob Agents Chemother. 2003; 47: 956-64.

45. Pic E, Burgain A, Sellam A. Repurposing the anthelminthic salicylanilide oxyclozanide against susceptible and clinical resistant *Candida albicans* strains. Med Mycol. 2019; 57: 387-90.

46. Qiao J, Sun Y, Gao L, He C, Zheng W. Lonafarnib synergizes with azoles against *Aspergillus* spp. and *Exophiala* spp. Med Mycol. 2018; 56: 452-7.

47. Rebong RA, Santaella RM, Goldhagen BE, Majka CP, Perfect JR, Steinbach WJ, et al. Polyhexamethylene biguanide and calcineurin inhibitors as novel antifungal treatments for *Aspergillus* keratitis. Invest Ophthalmol Vis Sci. 2011; 52: 7309-15.

48. Rodríguez MA, Cabrera G, Godeas A. Cyclosporine A from a nonpathogenic *Fusarium oxysporum* suppressing *Sclerotinia sclerotiorum*. J Appl Microbiol. 2006; 100: 575-86.

49. Rossato L, Venturini TP, Azevedo MI, Santurio JM, Alves SH. *In vitro* activity of immunosuppressive agents against *Cryptococcus neoformans*. Enferm Infecc Microbiol Clin (Engl Ed.) 2020; S0213-005X(20)30308-6.

50. Schmidt S, Hogardt M, Demir A, Röger F, Lehrnbecher T. Immunosuppressive compounds affect the fungal growth and viability of defined *Aspergillus* species. Pathogens. 2019; 8: 273.

51. Schwarz P, Schwarz PV, Felske-Zech H, Dannaoui E. *In vitro* interactions between isavuconazole and tacrolimus, cyclosporin A or sirolimus against Mucorales. J Antimicrob Chemother. 2019; 74: 1921-7.

52. Shinde RB, Chauhan NM, Raut JS, Karuppayil SM. Sensitization of *Candida albicans* biofilms to various antifungal drugs by cyclosporine A. Ann Clin Microbiol Antimicrob. 2012; 11: 27.

53. Shirazi F, Kontoyiannis DP. The calcineurin pathway inhibitor tacrolimus enhances the *in vitro* activity of azoles against Mucorales via apoptosis. Eukaryot Cell. 2013; 12: 1225-34.

54. Singh N, Heitman J. Antifungal attributes of immunosuppressive agents: new paradigms in management and elucidating the pathophysiologic basis of opportunistic mycoses in organ transplant recipients. Transplantation. 2004; 77: 795-800.

55. Singh K, Sun S, Vézina C. Rapamycin (AY-22,989), a new antifungal antibiotic. IV. Mechanism of action. J Antibiot (Tokyo). 1979; 32: 630-45.

56. Singh-Babak SD, Shekhar T, Smith AM, Giaever G, Nislow C, Cowen LE. A novel calcineurin-independent activity of cyclosporin A in *Saccharomyces cerevisiae*. Mol Biosyst. 2012; 8: 2575-84.

57. Spader TB, Ramírez-Castrillón M, Valente P, Alves SH, Severo LC. *In vitro* interactions of amphotericin B combined with non-antifungal agents against *Rhodotorula mucilaginosa* strains. Mycopathologia. 2019; 184: 35-43.

58. Steinbach WJ, Schell WA, Blankenship JR, Onyewu C, Heitman J, Perfect JR. *In vitro* interactions between antifungals and immunosuppressants against *Aspergillus fumigatus*. Antimicrob Agents Chemother. 2004; 48: 1664-9.

59. Steinbach WJ, Singh N, Miller JL, Benjamin DK, Schell WA, Heitman J, et al. *In vitro* interactions between antifungals and immunosuppressants against *Aspergillus fumigatus* isolates from transplant and nontransplant patients. Antimicrob Agents Chemother. 2004; 48: 4922-5.

60. Sugita T, Tajima M, Ito T, Saito M, Tsuboi R, Nishikawa A. Antifungal activities of tacrolimus and azole agents against the eleven currently accepted *Malassezia* species. J Clin Microbiol. 2005; 43: 2824-9.

61. Sun S, Li Y, Guo Q, Shi C, Yu J, Ma L. *In vitro* interactions between tacrolimus and azoles against *Candida albicans* determined by different methods. Antimicrob Agents Chemother. 2008; 52: 409-17.

62. Thakur M, Revankar SG. *In vitro* interaction of caspofungin and immunosuppressives against agents of mucormycosis. J Antimicrob Chemother. 2011; 66: 2312-4.

63. Tome M, Zupan J, Tomičić Z, Matos T, Raspor P. Synergistic and antagonistic effects of immunomodulatory drugs on the action of antifungals against *Candida glabrata* and *Saccharomyces cerevisiae*. PeerJ. 2018; 6: e4999.

64. Uppuluri P, Nett J, Heitman J, Andes D. Synergistic effect of calcineurin inhibitors and fluconazole against *Candida albicans* biofilms. Antimicrob Agents Chemother. 2008; 52: 1127-32.

65. Venturini TP, Al-Hatmi AMS, Rossato L, Azevedo MI, Keller JT, Weiblen C, et al. Do antibacterial and antifungal combinations have better activity against clinically relevant *Fusarium* species? *In vitro* synergism. Int J Antimicrob Agents. 2018; 51: 784-8.

66. Wibawa T, Nurrokhman L, Baly I, Daeli PR, Kartasasmita G, Wijayanti N. Cyclosporine A decreases the fluconazole minimum inhibitory concentration of *Candida albicans* clinical isolates but not biofilm formation and cell growth. Trop Biomed 2015; 32: 176-82.

67. Worasilchai N, Chindamporn A, Plongla R, Torvorapanit P, Manothummetha K, Chuleerarux N, et al. *In Vitro* susceptibility of Thai *Pythium insidiosum* isolates to antibacterial agents. Antimicrob Agents Chemother. 2020; 64: e02099-19.

68. Zhang J, Tan J, Yang L, He Y. Tacrolimus, not triamcinolone acetonide, interacts synergistically with itraconazole, terbinafine, bifonazole, and amorolfine against clinical dermatophyte isolates. J Mycol Med. 2018; 28: 612-6.
